# Multivariate Screening and Automated Clustering of Macrophage Immunoreactome to Nanoparticles and Photothermal Therapy

**DOI:** 10.1002/advs.202405860

**Published:** 2025-06-26

**Authors:** Sonia Becharef, Léa Jabbour, Nassima Bekaddour, Giulio Avveduto, Nathalie Luciani, Gautier Laurent, Rana Bazzi, Edouard Alphandery, Stéphane Roux, Amanda K. A. Silva, Kelly Aubertin, Jean‐Philippe Herbeuval, Florence Gazeau

**Affiliations:** ^1^ Université Paris Cité, NABI CNRS UMR8175, INSERM U1334 Paris France; ^2^ Nanobacterie Paris France; ^3^ Université de Franche‐Comté Chrono‐environnement, UMR 6249 CNRS/UFC Besançon 25030 France; ^4^ Université Paris Cité LCBPT CNRS, UMR8601 Team Chemistry & Biology, Modeling & Immunology for Therapy Paris France

**Keywords:** immunotherapy, macrophages, nanoparticles, photothermal therapy

## Abstract

Immunotherapy aims to control the immune system against diseases such as cancer or infections. Nanotechnology is part of the armamentarium to reprogram the immune system in a spatially and temporally controlled manner. However, predicting immune responses using high‐throughput tests is challenging due to the immunoreactome's complexity and plasticity. This work presents a fast, machine learning‐assisted predictive assay to classify the multifactorial immune responses to any kind of treatments. Engineered human THP‐1 monocytes differentiated and polarized into M0, M1, and M2 macrophages are used to monitor nuclear factor Kappa B (NF‐kB) and interferon regulatory factor (IRF) pathway activations and gene expression profile in response to metallic nanoparticles (NPs), activated or not by light to induce photothermal therapy (PTT). Principal component analysis (PCA) reveals distinct responses to nanoparticles and the reprogramming toward inflammatory macrophage triggered by PTT. Gold‐iron oxide nanoflowers and magnetosomes per se favor polarization toward M2 profile, while light activation shifts this M2‐like macrophages toward an M1 phenotype. These findings, confirmed on human blood derived monocytes shed light on the intricate immunomodulatory effects of nanoparticles and PTT on macrophage behavior and provide a basis for an adaptable screening method for the predictive design of therapeutic strategies for immunotherapy in cancer and other diseases.

## Graphical abstract

Predicting immune responses to treatments, including nanomedicines, with high throughput test is challenging due to the immunoreactome's complexity and plasticity. A predictive screening platform integrating pathway activation and gene profiling in macrophages has been developed as a versatile tool for designing effective immunotherapies. Automated clustering reveals switchable macrophage reprogramming from M2‐to‐M1 under photothermal therapy activating gold or iron oxide nanoparticles.

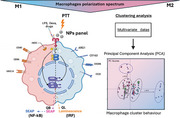

## 1 Introduction

The capacity to predict, control, and modulate the immune system in response to threats and pathological conditions is currently one of the most challenging and promising objectives of modern medicine. Acute inflammation, which normally leads to recovery from infection or tissue repair in the case of injury, can progress, when not properly phased by stimuli and checkpoints, to chronic inflammation, tissue destruction, remodeling, fibrosis, and even favor cancer progression. Immunomodulation is a therapeutic intervention that enhances or represses the body's defense mechanisms. Immunotherapy is revolutionizing the management of cancer, helping our immune system to recognize and fight malignant cells by stimulating the so‐called inflammatory “hot” tumor microenvironment (TME).

To progress in the control of immunity, immunotherapeutic approaches have rapidly expanded its armamentarium with numerous small molecules drugs as well as nanosystems that can activate innate and/or adaptive immune responses and show high affinity to cross‐talk with the immune system.^[^ ^1^, ^2^ ^]^ The remote activation of nanosystems that are responsive to light, magnetic fields, or other physical stimuli, can potentially offer additional spatial and temporal control over the targeted modulation of immunity in cancer, autoimmune, or inflammatory diseases.^[^ ^3^, ^4^ ^]^ However, given the complexity of the immune response to multiple environmental stimuli, it remains difficult to predict the immunomodulatory properties of a given treatment in a specific pathological context. There is a lack of in vitro immunomodulatory assays and companion analysis protocols that can embrace the broad spectrum of immune reactivity to compare drug candidates and allow for rational Artifical Intelligence‐assisted drug design and selection. In vitro high‐throughput high‐content screening methods combined with machine learning approaches, could help predict multifactorial effects of therapeutic interventions and drive immunomodulatory science in vivo, accelerating the development of novel and more precise immunotherapeutic approaches.

In this study, we propose a combination of versatile high‐content screening tests with a simple statistical analytic approach to appraise the immunomodulatory properties on macrophage functions of drugs or nanosystems, activated or not with external fields. Our test is designed to embrace a broad range of macrophage phenotypes and functions, and to elucidate, by means of principal components analysis (PCA), how some specific nanoparticle treatments, coupled or not to photothermal activation, can shift or reprogram the macrophage immunoreactome in a precise way. This screening model can identify combinatory treatments to finely and timely control macrophage reactivity in different pathological contexts, including solid tumors and inflammatory disorders.

## 2 Results and Discussion

### 2.1 Building up a Screening Model to Assess Macrophage Response Patterns to Different Treatments

Monocytes and macrophages are innate immune cells involved in multiple pathophysiological processes, including (i) clearance of dead cells and recognition of pathogens such as bacteria or viruses via pattern recognition receptors, (ii) increased proliferation to eliminate a large diversity of pathogens, (iii) recruitment of other immune cells by functioning as antigen‐presenting cells, leading to an indirect activation of immune effectors such as T cells, and (iv) regulation of inflammation through the production of pro‐ or anti‐inflammatory cytokines.^[^ ^5^ ^]^ Macrophages display high plasticity, allowing them to adapt their phenotypes depending on context, environmental cues, and response to stimuli, including NPs.^[^ ^6^, ^7^ ^]^ In the past decades, intensive phenotyping of macrophage lineages in different contexts has revealed distinctive macrophage functional subsets and/or patterns of activity.^[^ ^8^ ^]^ In vitro models of monocyte‐derived macrophages have been extensively used as surrogates to assess in vivo macrophage function, focusing on schematized subsets with different polarization M0, M1, and M2. Monocytes are commonly differentiated to produce naive macrophages (M0) and then polarized into M1 and/or M2 macrophages with opposite functional patterns. M1 macrophages exhibit proinflammatory effects through the secretion of proinflammatory cytokines such as TNFα (Tumor Necrosis Factor alpha), IL (Interleukine) ‐6, IL‐12, IL‐23, or IFN‐γ (Interferon gamma). The microbicidal and phagocytosis machinery of M1 macrophages allows them to participate to the clearing of acute infections and elimination of xenobiotics. However, excessive or prolonged M1 polarization can lead to tissue injury and contribute to pathogenesis. In contrast, M2 macrophages are involved in the regulation of the immune response to restore homeostasis within tissues through the secretion of multiple anti‐inflammatory cytokines such as IL‐10 and presents regenerative properties. Nevertheless, M2 macrophages cover a continuum of phenotypic and functional properties. For example, specific M2 program induced by bacterial infection can lead to a chronic evolution of infectious diseases.^[^ ^9^ ^]^ In tumors, monocyte‐derived macrophages are recruited from the blood vessels to infiltrate the tumor. These cells are reconfigured by the tumor microenvironment into tumor‐associated macrophages (TAM) that mostly exhibit a M2‐like phenotype.^[^ ^10^ ^]^ Those macrophages secrete immunosuppressive signals that block the Th1‐type immune activity, losing the immune control of cancer cells, promoting tumor growth, angiogenesis, matrix remodeling, and favoring resistance to treatments.^[^ ^10^ ^]^ Therefore the M2 macrophage subset has emerged as a promising target for cancer therapy, and the induction of a shift in polarization from M2 to M1 macrophages holds significant potential in advancing anti‐tumor therapeutic strategies.^[^ ^11^, ^12^ ^]^ In essence, the ability of the monocyte/macrophage system to adapt to various environmental signals, challenges the notion of fixed tissue‐ and response‐specific differentiation pathways and calls for a continuously evolving array of functional patterns among diverse populations. Therefore, an in vitro screening model to assess the multifactorial effects of various treatments should encompass the entire spectrum of macrophage response patterns.

**Scheme 1:**
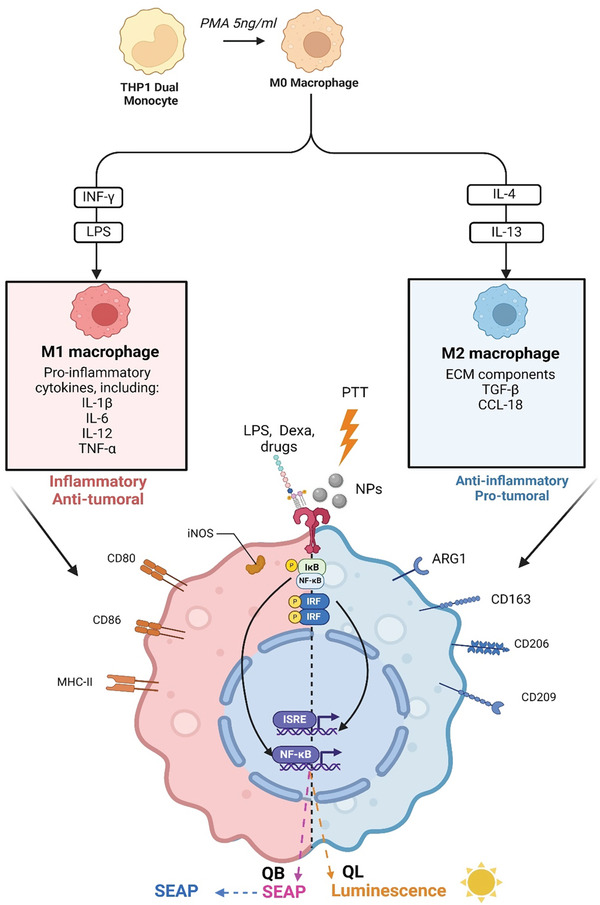
Schematic illustration of a screening test platform to predict the effects of different treatments on macrophage polarization and inflammation in vitro. PMA: phorbol 12‐myristate 13‐acetate; Dexa: dexamethasone; IRF: interferon regulatory factor pathway; NF‐κB: nuclear factor kappa B pathway; SEAP: secreted embryonic alkaline phosphatase. QB: QUANTI‐Blue, QL: QUANTI‐Luc, TLR: toll‐like receptor; NPs: nanoparticle, ISRE: interferon stimulated response element. Scheme created with BioRender.com.

To achieve this objective, we used monocyte‐derived macrophages that were polarized into M0, M1, and M2 phenotypes as the recipient cells for nanoparticles, drugs, and light exposure. We then examined the resulting manifestation of various unique functional phenotypes using multivariate statistical analysis (**Scheme** **1**). This rapid and straightforward test is designed as a screening tool to offer a snapshot of the inflammation and polarization status of macrophages in vitro under various treatments. To validate the assay, metallic iron oxide and gold nanoparticles (NPs) and gold salts were tested with or without photoactivation. To achieve this, we took advantage of THP‐1 Dual,^[^ ^13^ ^]^ a commercial human monocytic cell line genetically modified through transfection to specifically monitor the nuclear factor Kappa B (NF‐κB) signal transduction pathway and interferon regulatory factor (IRF), both of which are involved in inflammation. For the detection of NF‐κB pathway activation, a reporter construct expressing “secreted embryonic alkaline phosphatase” (SEAP) gene is activated via PRR (pattern recognition receptors) or TLR (Toll‐like receptor) receptor under the control of a promoter activated by the transcription of NF‐κB pathway genes. SEAP secreted into the medium is thus quantified by measuring absorbance using a detection reagent. For the detection of IRF pathway, IFN‐stimulated response element (ISRE) genes are expressed under the control of the Interferon‐Stimulated Gene 54 (ISG54) promoter activated in response to external stimulation. ISRE expression is linked to a luciferase reporter construct (Scheme 1). Luciferase production thus allows measurement of IRF pathway activation using a detection reagent.

**Figure 1:**
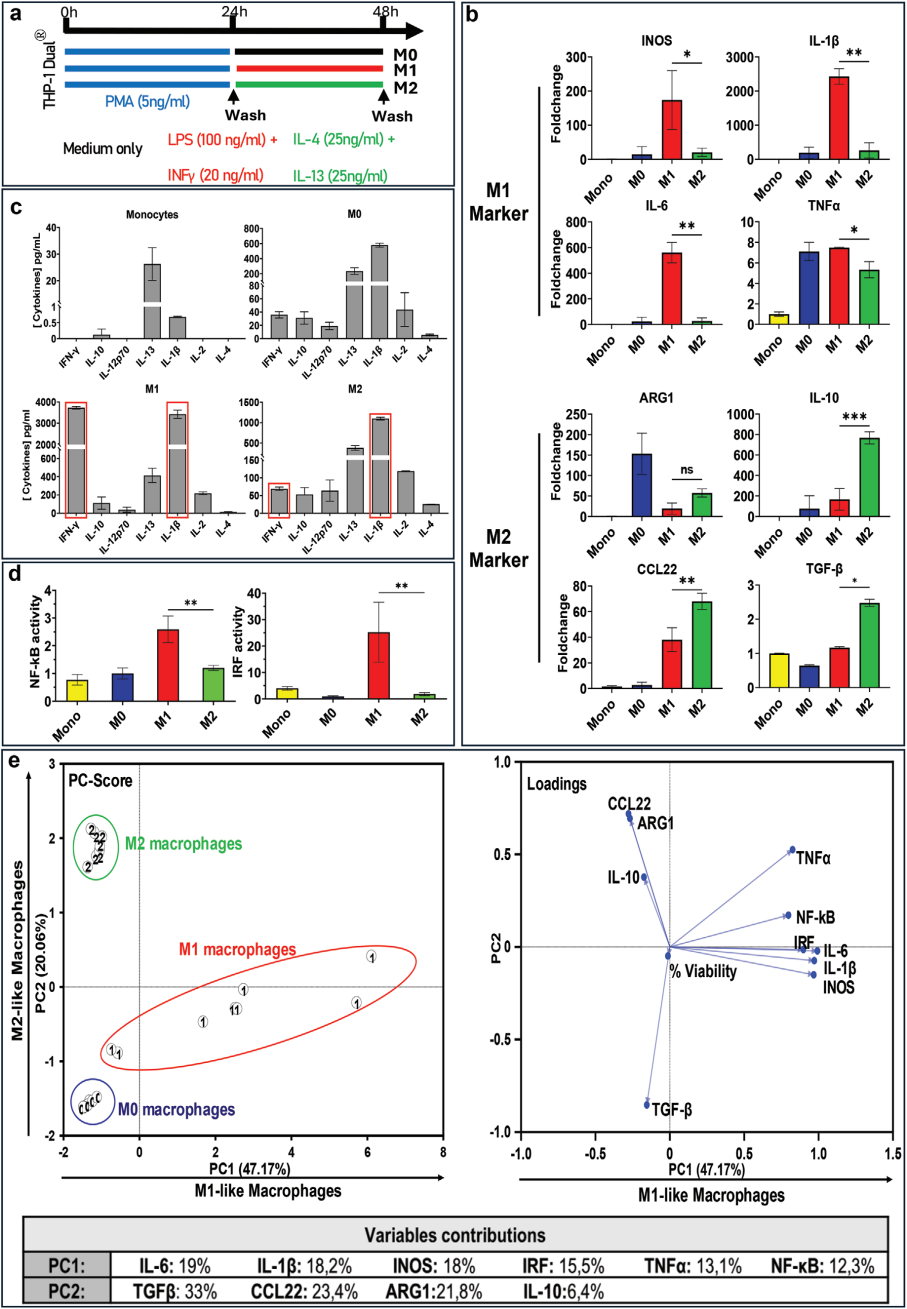
Validation of the differentiation and polarization protocol on THP‐1 Dual cell line. a) Protocol of polarization used. b) Relative mRNA expression of M1 and M2 macrophage markers was assessed in monocytes and M0, M1, and M2 macrophages using qPCR. Analysis was conducted on untreated samples and normalized to the RPLP0 housekeeping gene. Results are presented as foldchange based on monocytes and expressed as mean ± SD. *N* = 3. c) Quantification of human proinflammatory cytokines using MSD multiplex ELISA. Samples were collected from supernatant of THP‐1 Dual monocyte and differentiated macrophages into M0, M1, and M2 after 48 h in culture. d) NF‐κB and IRF activation of THP‐1 Dual monocytes and macrophages. NF‐κB and IRF signaling pathways were quantified using QUANTI‐Luc and QUANTI‐Blue, respectively. Data were normalized into foldchange based on M0 macrophages. e) The principal component analysis (PCA) was carried out on differentiated macrophages, utilizing the methodology described in this study and based on data from b and d. qPCR and pathway activation data were normalized on M0 macrophages. PCA automatically identified three distinct clusters representing M0, M1, and M2 differentiated macrophages (shows as 0, 1, and 2, respectively on the PCA scatter plot (PC scores, left)), thereby validating the reliability of our differentiation protocol. The loading plot (right) refers to the contributions of the different features into the two first PCs. The contributions are detailed in the variable contributions table. The percentage next to the PCs axis represents the percentage of the total variance of the data explained by each principal component individually. Results are shown as the mean ± SD, *N* = 3. Results of univariate statistical analysis are illustrated by ^*^ *p* < 0.05, ^**^ *p* < 0.01, or ^***^ *p* < 0.001.

The first step was to validate whether previously defined differentiation protocol could induce polarization into M1 and M2 macrophages of the THP‐1 Dual cell line. The THP‐1 Dual monocytes were first treated with phorbol 12‐myristate 13‐acetate (PMA) for 24 h to induce M0 macrophage differentiation. Subsequently, the cells were washed and exposed to different cocktails, consisting of lipopolysaccharides (LPS) + IFN‐γ or IL‐4 + IL‐13, to polarize toward M1 and M2 macrophages, respectively, over a 24‐h period (**Figure** **1**a). Morphologically, THP‐1 Dual‐derived M0 macrophages exhibited a rounded and compact shape, while M2 macrophages displayed a tendency to branch toward neighboring cells. In contrast, M1 macrophages displayed a distinct fusiform appearance compared to M0 macrophages (Figure S1, Supporting Information). These observable phenotypic variations provided initial evidence of successful differentiation. Furthermore, qPCR analysis showed that genes associated with M1 characteristics (iNOS, IL‐1β, IL‐6, and TNFα) were expressed at higher levels in THP‐1 Dual‐derived M1 macrophages than in monocytes, M0, or M2 macrophage subsets. In contrast, genes known to present an elevated expression in M2 macrophages (ARG1, IL‐10, CCL22, TGF‐ β) were highly expressed in THP‐1 derived M2 macrophages (Figure 1b). The differentiation protocol was also assessed by analyzing the release of cytokines. Monocytes exhibited minimal proinflammatory cytokines secretion, indicative of their noninflamed status. In comparison, M0 macrophages demonstrated higher levels of proinflammatory cytokines secretion compared to monocytes, consistent with the inherently more inflammatory nature of macrophages compared to monocytes. Finally, the results showed that THP‐1 derived M1 macrophages secreted significantly higher levels of IFN‐γ and IL‐1β than the M2 counterparts (Figure 1c). These findings are consistent with the established understanding that M1 macrophages exhibit a more inflammatory profile than M2 macrophages.^[^ ^8^ ^]^ The behavior of differentiated THP‐1 Dual macrophages appeared to mimic that of primary macrophages. To further investigate the inflammatory pathways specifically expressed in THP‐1 Dual cells, namely NF‐κB and IRF, the activation levels of these two transcription factor families, key components of inflammation signaling, were examined. The results showed in Figure 1d are the one at 48 h and indicate that the activation of these two pathways increased in the M1 polarization state compared to that in both M0 and M2 cells. These findings confirm the successful differentiation of THP‐1 Dual monocytes to M0 and then polarization to M1 and M2 phenotypes, with heightened inflammatory profile in M1 compared to their M0 and M2 counterparts, and with distinctive M1 and M2 characteristics.

Based on the multiple features that were measured to characterize each macrophage subpopulation, namely the cell metabolic activity (viability), the gene expression levels and the activation level of NF‐κB and IRF inflammatory pathways, we performed a multivariate analysis by principal component analysis (PCA) to present the outcomes of the differentiation/polarization protocol in an integrated way with reduced dimensionality. In Figure 1e, the graph displays the PC score of the THP‐1 Dual derived macrophages. Percentage next to the PC axis represents the percentage of total the total variance of the data explained by each PC individually. This percentage is depicted in each PCA graph, and the explanation provided is consistent throughout; therefore, it will not be repeated in subsequent descriptions. The abscissa, or horizontal axis (labeled PC1), depicts a gradient of macrophage polarization toward an M1‐like profile, with PC1 explaining 47.17% of the data variance. This is corroborated by the adjacent loading graph, which includes a variety of proinflammatory genes and cytokines such as IRF, NF‐κB, iNOS, and IL‐1β. The ordinate, or the vertical axis (labeled PC2), represents a polarization gradient toward an M2‐like profile explaining 20.06% of the data variance and characterized by genes such as CCL22, ARG1, and IL‐10, as depicted in the loading graph. In all PCA graphs, THP‐1 Dual‐derived macrophages M0, M1, and M2 according to the above protocol will consistently be labeled as “0”, “1”, and “2”, respectively. As expected, three primary clusters are visible: one at the bottom left corresponding to M0 cells, another at the top left representing M2 cells, and an expanded cluster at the bottom right characterizing M1 macrophages. These cells exhibit significant polarization plasticity and variability, with some cells remaining closer to the M0 cell state and others displaying an extreme M1 phenotype on the far right. This first PCA confirms that the set of variables and features that were quantified is sufficient to cluster the nominal M1 and M2 polarization of macrophages, also clearly distinguished from naïve THP‐1 Dual derived M0.

**Figure 2:**
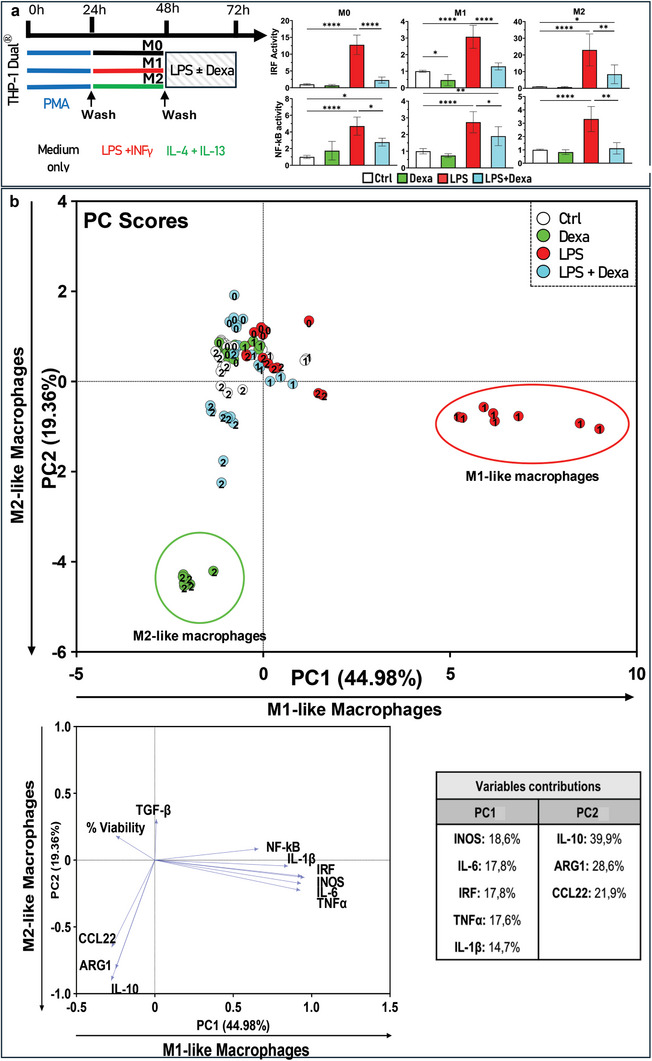
a) Polarization protocol employed, and control treatments used to modulate macrophage polarization and NF‐κB or IRF activity. The macrophages were treated with two different agents: Lipopolysaccharide (LPS), which acts as an inflammatory agent, triggering Toll‐like receptors (TLRs) and prompting a proinflammatory response; dexamethasone, which was used as an anti‐inflammatory control. Combination of LPS and dexamethasone was used to evaluate the protective effects of dexamethasone against inflammation. b) PCA was carried out to uncover the immunomodulatory effects of differentiated macrophages, with these additional control treatments considered for examination. Scatter plot (PC scores, top) exhibited three clusters, two of them corresponding to extremely polarized macrophages (i.e., M1 macrophages treated with LPS and M2 macrophages treated with Dexa). The third cluster regroup controls and macrophages treated with inflammatory agent inducing opposing effect to the polarization. The loading plot (bottom) refers to the contributions of the different features into the two first PCs. The contributions are detailed in the variable contributions table. Results of univariate statistical analysis are illustrated by ^*^ *p* < 0.05, ^**^ *p* < 0.01, or ^***^ *p* < 0.001.

To establish a pertinent multivariate analysis for characterizing the multifaceted immunomodulatory impact of various treatments, we utilized well‐known pro‐ and anti‐inflammatory agents as control measures. LPS serves as an inflammatory agent to induce TLR4 activation, while dexamethasone, a synthetic corticosteroid commonly used as an anti‐inflammatory agent, suppresses the expression of inflammatory mediators. Finally, a combination of dexamethasone and LPS is used to evaluate the protective effects of dexamethasone on LPS‐induced inflammation. Above treatments were applied on culture of M0, M1, and M2 THP‐1 derived macrophages. The impact of control treatments is illustrated in **Figure** **2**a that depicts the activation of the IRF and NF‐κB pathways. Treatment with LPS appears to activate both pathways across different cell lines, while dexamethasone displays a modest inhibitory effect on IRF pathway activity exclusively in M1 macrophages and appears ineffective in modulating NF‐κB activity within these proinflammatory cells. This observation may be attributed to the pronounced activation state of M1 macrophages induced by our protocol. In instances where inflammation is already established within the M1 proinflammatory profile, dexamethasone encounters challenges in reducing inflammation. Moreover, to identifying the optimal timing for observing the activation of both the NF‐κB and IRF pathways on THP‐1 Dual monocytes and macrophages, we performed a monitoring of NF‐kB and IRF pathways over time at intervals of 3, 24, and 48 h following the different treatment. The findings suggest that M1 macrophages treated with dexamethasone exhibit a notable protective activity against pathway activation at the 24‐h mark, which diminishes by the 48‐h time point (Figures S2–S4, Supporting Information). However, combined treatment with dexamethasone and LPS demonstrates an intermediate effect, potentially due to dexamethasone protective role in inflammatory conditions provoked by LPS.

PCA results in Figure 2b depict the impact of these opposite control treatment in the macrophage polarization. The horizontal axis (PC1) illustrates a gradient of macrophage polarization toward an M1‐like profile while the vertical axis (PC2) represents a gradient of M2‐like polarization extending from top to bottom. Two additional distinct populations were discernible in the PCA score plot. A red cluster to the right of PC1 corresponds to M1 cells treated with LPS, whereas a green cluster positioned at the left of PC1 and bottom of PC2 corresponds to M2 cells treated with dexamethasone. These clusters demonstrate the capacity of LPS and dexamethasone to drastically shift the polarization status of macrophages along the gradients principally defined by PC1 and PC2. Examination of loading vectors revealed that variables associated with the red cluster included IRF, iNOS, IL‐6, IL‐1 β, TNFα, and NF‐kB, which are markers of M1. Conversely, the variables associated with the green cluster included CCL22, ARG1, and IL‐10, indicating M2‐like characteristics. On the PC2 axis from top to bottom, we observe successively the clusters of M0, then M1 and M2 macrophages, treated or nontreated, while on the PC1 axis, from left to right, we observe M2, M0, and then M1 subsets. Treatment with LPS tends to shift all populations to the right, with the most significant effect observed in the M1 subset. In contrast, treatment with dexamethasone has a prominent effect only on M2 subset. Interestingly, the combination treatment of LPS and dexamethasone mitigates the effect of dexamethasone on M2, and of LPS on M1, on PC2 and PC1 axis, respectively, whereas it tends to shift the M0 to the top. This clear and consistent separation of cellular profiles demonstrates the reactivity of M1 and M2 THP‐1 Dual derived macrophage to LPS, dexamethasone, or combined treatment. These results validate our screening model based on THP‐1 Dual monocytes coupled to PCA to investigate the macrophage plasticity and its regulation by external stimuli.

In the following experiments, we use this novel methodological approach leveraging PCA to appraise the immunomodulatory effects of nanoparticles and metallic salts, with or without photoactivation, that have been proposed as innovative treatments strategies, particularly in the field of cancer.^[^ ^14^, ^15^ ^]^

### 2.2 Broad Spectrum Impact of Various Gold and Iron Oxide Nanoparticles and Gold Salts on Macrophage Polarization and Inflammatory Status

To explore the full potential of our screening model in assessing macrophage responses, we applied it to evaluate the broad‐spectrum impact of a selection of gold and iron oxide nanoparticles as well as gold salts. These nanoparticles were specifically chosen for their relevance in clinical and preclinical applications including imaging contrast agents^[^ ^16^, ^17^ ^]^ drug carriers, photo‐ or magnetically activable nanosystems,^[^ ^18^ ^]^ anti‐bacterial agents, or metal supplements. Despite their rapid uptake by monocyte/macrophage or their internalization by TAMs within the tumor microenvironment, the specific influence of NPs on macrophage physiology remains unclear and probably tissue dependent.^[^ ^19^ ^]^ Given their extensive application in cancer diagnosis, therapy, and anaemia^[^ ^20^ ^]^ as well as inflammatory diseases treatment, studies deciphering the broad spectrum of macrophage modulation by NPs should be of significant interest to predict in vivo response.

**Figure 3:**
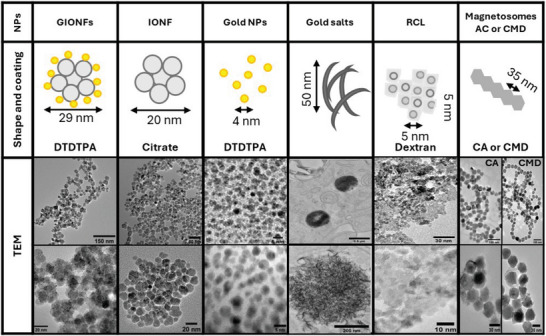
Description of NPs used in this study. Gold‐decorated Iron Oxide Nanoflowers (GIONF) consist of citrate‐coated 20 nm multicore iron oxide nanoflowers (IONF) decorated with 2–5 nm gold NPs coated with dithiolated diethylenetriamine pentaacetic acid (DTDTPA). Sodium aurothiomalate was used to treat THP‐1 Dual derived M0 macrophages resulting in the formation of intralysosomal nanoassemblies of tiny gold 2 nm nanoclusters, forming curved lashes, also called aurosomes. RCL NPs are multicore magnetic plates embedded in a dextran (H(C_6_H_10_O_5_)_x_OH) 40 kDa matrix. Magnetosomes isolated from magnetotactic bacteria were coated with carboxymethyl dextran (CMD) or citric acid (CA). Transmission electron microscopy (TEM) was employed to obtain images of the NPs deposited on the grid. In case of Au salts, TEM pictures show 70 nm slices of THP‐1 macrophages (M0) treated with Au salts for 24 h and embedded in resin. The size distribution of each type of nanostructures has been quantified from TEM pictures and is shown in Figure S6 (Supporting Information).

In this study, we take advantage of the above methodological approach to comprehensively assess the broad‐spectrum impact of several NPs. Seven NPs were chosen based on their differences in shape, materials (gold and iron oxide), coating and size distribution (**Figure** **3**) and their promising therapeutic applications. Citrate coated iron oxide nanoflowers (IONFs) are monocrystalline 20–25 nm multicore maghemite (γ‐Fe_2_O_3_) superparamagnetic NPs with unique magnetic properties that make them outstanding contrast agents for MRI and one of most efficient magnetic nanostructures known to generate hyperthermia under alternating magnetic field.^[^ ^21^ ^]^ They have been also reported as efficient photothermal agents under near‐infrared (NIR) light (808 nm) irradiation in preclinical models of solid tumors, especially when IONF were decorated with ultrasmall gold NPs measuring 2–5 nm and coated with dithiolated diethylenetriaminepentaacetinanoparticles (Au@DTDTPA), forming the so‐called gold iron oxide nanoflowers (Au@DTDTPA@IONF or GIONF)^[^ ^22^, ^23^ ^]^ The efficacy of GIONF as PTT agents for softening and shrinking cholangiocarcinoma human tumor xenografts was demonstrated and their immunomodulatory properties were investigated in a syngeneic model of triple negative breast cancer.^[^ ^23^ ^]^ Furthermore, ultrasmall Au@DTDTPA also function as radiosensitizing nanoagents with the aim of enhancing the efficacy and targeted delivery of radiotherapy. Additionally, these nanoparticles have been envisioned to support image‐guided therapy and promote renal clearance.^[^ ^24^, ^25^, ^26^ ^]^ The selection of these three types of nanoparticles (IONF, GIONF, and Au@DTDTPA) was primarily driven by their multifaceted applications coupled with their controlled biodegradability^[^ ^23^ ^]^ in cellular environments.^[^ ^27^ ^]^ In addition, sodium aurothiomalate is a gold salt that has been used for decades as bactericides and anti‐inflammatory agents for the treatment of rheumatoid arthritis.^[^ ^28^ ^]^ Importantly it has been shown that Au salts can be rapidly internalized by cells and compartmentalized within lysosomes, where they undergo bio‐mineralization in lysosomal compartment, forming first three‐dimensional (3D) arrangement of tiny gold nanoclusters and ultimately 2D shaped nanostructures reminiscent of curved leaves. Such gold rich lysosomes named “aurosomes,” were observed in patients treated with Au salts for months. These intracellular nanostructures exhibit long term bio‐persistence, sparking interest in the study of gold‐based nanomaterials for biomedical applications and potential photosensitivity. Notably, we recently showed that the later stages of intralysosomal degradation of gold nanoparticles appear to coincide with this gold biomineralization process starting from Au salts, highlighting a common mechanism of gold metabolism^[^ ^28^, ^29^ ^]^ Using our screening approach, we treated THP‐1 Dual derived macrophages (M0) with aurothiomalate for 24 h and observed aurosomes formation, consisting of 3D assemblies of curved lashes within membrane bound endo‐lysosomes (Figure 3). These intracellular gold nanostructures evolve after 7 days into larger and more defined 2D curved leaves, as previously observed in treated patient's cells (Figure S5, Supporting Information). These aurosomes piqued attention as intriguing biogenic platform for selective targeting and therapeutic administration due to their potential optical properties resulting from the gold quantum confinement effect and light‐absorbing capabilities.^[^ ^30^ ^]^ In the context of the tumor microenvironment or inflammatory diseases, research focusing on aurosomes immunomodulation of the microenvironment through macrophage polarization or inflammation, with and without light activation, holds promise for our understanding of their immunomodulatory effects and potential in therapy.

Another category of iron‐based NPs investigated in this study are bacterial magnetosomes manufactured by the company Nanobactérie. These NPs are nanostructures that are naturally found inside magnetotactic bacteria named *Magnetospirillum magneticum* strain AMB‐1. Magnetosomes are composed of aligned magnetic particles surrounded by a bacterial membrane. They typically have a size range of 30–37 nm and are primarily composed of magnetite (Fe_3_O_4_) or maghemite (γ−Fe_2_O_3_). Following extraction from bacteria to render them biocompatible and nontoxic, magnetosomes are coated with substances such as carboxymethyl dextran (CMD) or citric acid (AC).^[^ ^31^ ^]^ Magnetosomes exhibit interesting properties for magnetic and light‐induced hyperthermia. These nanoparticles are distinguished by their high crystallinity and chain‐like organization, endowing them with superior heating capabilities until the optimal temperatures of 41–50 °C under the influence of an alternating magnetic field. The surface coating of poly‐l‐lysine further enhances their stability and reduces toxicity risks. Notably, preclinical models demonstrated that magnetosomes completely eradicate tumors in mice, illustrating their effectiveness as a cancer treatment.^[^ ^32^ ^]^ Finally, clinical grade RCL iron oxide NPs, supplied by Resonant Circuits Limited, were used in our investigation. These RCL nanoparticles are currently under clinical evaluation for magnetic hyperthermia associated with standard of care chemotherapy to treat with locally advanced pancreatic cancer.^[^ ^33^, ^34^ ^]^ They belong to a class of multicore magnetic NPs characterized by the presence of multiple distinct magnetic cores, each enclosed within a nonmagnetic matrix that physically isolates them from adjacent cores.^[^ ^35^ ^]^ The magnetic cores are primarily composed of magnetite (Fe_3_O_4_) and/or maghemite (γFe_2_O_3_), whereas the non‐magnetic matrix comprises a low‐molecular‐weight polysaccharide dextran (H(C_6_H_10_O_5_)_x_OH), with an approximate molecular weight of 40 kDa.

**Figure 4:**
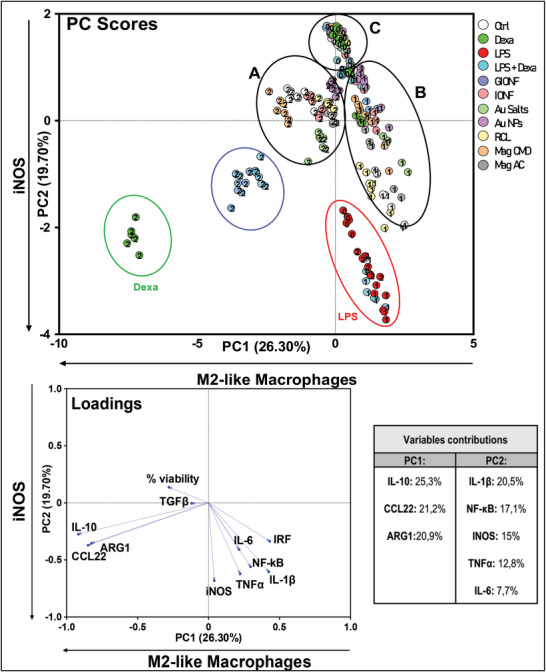
PCA was conducted on M0, M1, and M2 macrophages and exposed to various nanoparticles (NPs) and Au salts. LPS and Dexamethasone was used as pro and anti‐inflammatory control respectively. The figure presents scatter plot (PC scores, top) showing six clusters. The loading plot (bottom) refers to the contributions of the different features into the two first PCs. The contributions are detailed in the variable contributions table.

To assess the immunomodulatory effect of the different NPs and gold salt treatments, M0, M1, and M2‐like macrophages were treated for 24 h and the different descriptors characterizing macrophage polarization and inflammation status were measured and analyzed by PCA. This approach enables an integrated and high‐throughput evaluation of cellular responses to diverse treatments, in comparison to the standard dexamethasone and LPS treatments (**Figure** **4**). Importantly, exposure to NPs and Au salts was carried out at concentrations that were below the toxicity thresholds for macrophages, with the aim of reducing any potential confounding effects on gene expression or inflammation modulation resulting from cell death. The non‐toxic concentration was set to 5 µg mL^−1^, based on the measurements of the mitochondrial metabolic activity of cells as shown in Figure S7 (Supporting Information). Interestingly, when aggregating all the NPs and salt treatments to the “control” LPS and dexamethasone treatments, the two PC that emerged from the PCA were different from the PC with control treatments alone (Figure 2), showing distinct immunoreactome to the NPs. PC1 demonstrates a gradient of polarization toward M2‐like macrophages. PC2 suggests a gradient from anti‐inflammatory to proinflammatory properties principally via the expression of iNOS, IL‐1β and activation of NF‐κB pathway.

PCA reveals distinct clusters crossing these axes, each associated with specific treatments, highlighting the diversity of the immunological reactions to NPs and Au salts, showcasing the ability of our screening approach to distinguish diverse immunological reactions. For example, M2 treated with dexamethasone are found in the cluster outlined in green at the bottom left of the axes, signalling a deep polarization toward the M2‐like phenotype. In contrast, the cluster outlined in red at the bottom right encompasses sequentially M0, M2, and M1 treated with LPS, indicating a gradient of proinflammatory response and a shift to M1‐like phenotype. The clusters A, B, and C outlined in black, encompass respectively the M2, M1, and M0 cells treated with various NPs or non‐treated. M0 cluster “C” is located in the upper middle section displaying no polarization toward either M1 or M2‐like macrophages, nor any activation of the NF‐kB pathway or iNOS expression, which is consistent with the anticipated outcomes for M0 macrophages. Consistently, M2 cluster “A” and M1 cluster “B” are located on the left and right on the PC1 axis, respectively. The variability in the cellular responses to the different NPs can be observed within the clusters A, B, and C, demonstrating the nuanced responses captured by our screening test. Notably, none of the NPs drastically reprogram the polarization of macrophage subsets. However, in comparison to the untreated M1 and M2 controls, we observe that most of the NP's treatment, especially the gold NPs, gold salt, IONF, and magnetosomes AC tend to depolarize both M1 and M2 subsets, making them closer to M0. Interestingly we observed distinct behavior of GIONF and magnetosomes CMD, that shift M1 toward M0 cluster, but also displace M2 toward the cluster of M2 treated with dexamethasone. GIONF have the most prominent effect on M2 sharing a common cluster with M2 treated with LPS and dexamethasone. In conclusion, this analysis provides valuable insights into the correlation between macrophage inflammatory status and applied treatments, validating the strength of our screening approach in identifying immunomodulatory properties of the tested NPs.

### 2.3 Nanoparticles‐Mediated Immunomodulation of Macrophages is Differentially Reprogrammed by Photothermal Activation

In solid tumors, dense desmoplastic tumor presenting a stiff extracellular matrix are associated with poor prognosis due to resistance to conventional therapies. Therefore, novel strategies targeting ECM are envisioned to break down the physical barriers that restrain antibodies and immune cells to penetrate solid tumors and initiate immunogenic cell death.^[^ ^23^, ^36^ ^]^ Recent studies, including ours, showed that a less stiff ECM allows for the enhancement of immune cells infiltrating the tumor.^[^ ^37^ ^]^ In this context, NPs remotely activated by external NIR or alternating magnetic field can induce local and controlled mild hyperthermia in the tumor region.^[^ ^14^ ^]^ This controlled heating can elicit multifactorial effects on the TME, including destructuring, tumor softening,^[^ ^18^ ^]^ cell apoptosis or ferroptosis,^[^ ^38^, ^39^ ^]^ lysosomal disruption^[^ ^39^ ^]^ and potential shifting the immune environment from “cold” to “hot”.^[^ ^15^, ^22^ ^]^ NPs with different materials, sizes, shapes are often selected based on their light‐to‐heat energy conversion properties and biocompatibility^[^ ^40^ ^]^ however little is known on their immunomodulatory properties, especially when activated by an external field.^[^ ^22^ ^]^ As many preclinical tumor models rely on human xenograft in immunodeficient mice, the endogenous immune response to NPs and PTT remains poorly investigated, even if recent studies pointed out very different biodistribution and outcomes of NPs in immunocompetent models.^[^ ^36^ ^]^

**Figure 5:**
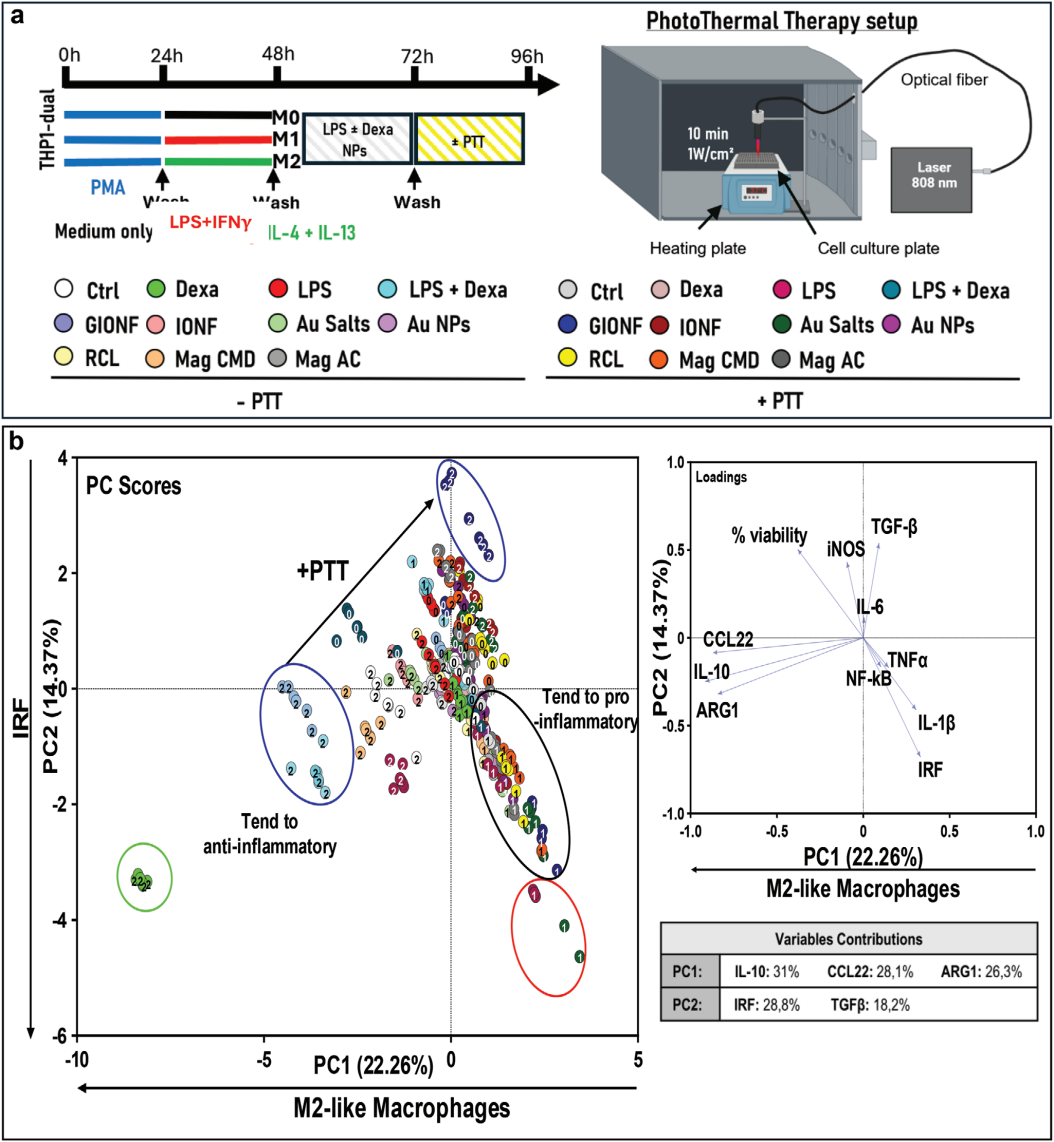
a) Protocol for polarization was employed with and without photothermal therapy (PTT), utilizing a PTT session at 808 nm for 10 min at a power density of 1 W cm^−^^2^. b) The two PCA graphs illustrate the impact of various nanoparticles (NPs) on macrophage polarization and inflammation status, with and without PTT activation.

To fill this gap, we used our screening model to study how NP‐mediated hyperthermia affects the macrophage inflammatory status and polarization (see Figures S8 and S9, Supporting Information, for the detailed results). As shown above, it was important to assess the immunoreactome to different NPs treatment first in absence of external activation. We next added a sub‐toxic exposure to NIR light (PTT) to evidence the specific effect of photoactivation on macrophages (**Figure** **5**a). As before, the viability of cells was evaluated to ensure that the observed effects were not caused by cell death due to NPs or photoactivation. A concentration of 5 µg mL^−1^ of NPs was chosen because it was found to be non‐toxic without PTT, but still led to some moderate cell death after light irradiation with the viability remaining above 70% (see Figure S7, Supporting Information). Additionally, we confirmed that the observed effects were due to nanoscale heating within the cells caused by photoactivation, rather than heating of the culture medium (see Figure S10, Supporting Information).

Upon examination of the PC score graphs (Figure 5b), the ordinate axis PC2 displays a gradient of IRF pathway activation from top to bottom. Simultaneously, the abscissa PC1 illustrates a gradient of M2‐like macrophage polarization from right to left. For each NPs treatment, results are indicated by dots with light color without PTT and dark color after PTT. Thus, one should pay attention to the shift from light to dark dots that emphasizes the outcome of PTT for specific NPs. As before, a distinct cluster outline in green encompasses M2 macrophages treated with dexamethasone. In contrast, a conspicuous cluster outlined in red comprised M1 cells treated with LPS but also M1 cells treated with gold salt and PTT, indicating a pronounced proinflammatory profile via IRF activation. M2 macrophages treated with NPs, without PTT, occupy the left side of PC1 axis, whereas M1 cells treated with NPs alone and with PTT are all located on the right side. The widespread cluster outlined in black contains M1 cells treated with NPs and PTT that align along a proinflammatory trajectory confirming the impact of NPs combined with PTT to enhance the inflammation state of the cells. Positions along this trajectory classify the NPs in descending order of their proinflammatory effect under light exposure: Au salt, GIONF, RCL, magnetosome CMD, IONF, Gold NPs, and magnetosome AC. Interestingly, the outcome of PTT on M2 macrophages treated with NPs is even more contrasted. PTT tends to shift the M2 clusters to the top right, sometimes beyond the M0 clusters, demonstrating M2 reprogramming into M1‐like macrophages with an increase in TGF‐β and iNOS expression. This reprogramming effect is the most pronounced for GIONF. As seen above, GIONF alone exhibit the same effect as the combination of LPS and dexamethasone. However, when exposed to light, M2 are reprogrammed to the cluster outlined in blue at the extreme position on the top right. Therefore, the intriguing pro‐M2 effect of GIONF alone is completely reversed by light activation inducing an overexpression of iNOS and TGF‐β and bypassing the activation of IRF.

The other NPs treatments when combined with PTT also induce a shift of M2 macrophages to the top right of the pattern. This effect was much less pronounced than for GIONF. Interestingly, M2 treated with NPs and PTT are located close to or beyond the cluster encompassing M0, which demonstrates the reversion of M2 polarization by PTT. On the other hand, in the case of M1 macrophages, the combination of NPs with PTT doesn't change the macrophage polarization. However, PTT induces a more pronounced M1‐like polarization and increased the inflammatory status of these cells by activating the IRF pathway.

This multivariate analysis and automated clustering of macrophage immunoreactome to the different NPs revealed the nuanced spectrum of macrophage responses and allows to compare, rank, and classify the NP's and PTT treatments as function of their immunomodulatory outcome. While this statistical analysis gives a very general and integrated view of the differential effects of NPs combined to light activation, it also revealed some NPs with unique behaviors, among which we focused on GIONF, magnetosomes CMD, and gold salts. In the following, we examine in more details the macrophage responses to these NPs.

### 2.4 GIONF: Photoactivable Cooperation of Gold and Iron Oxide to Reverse Macrophage Polarization and Inflammation Status

**Figure 6:**
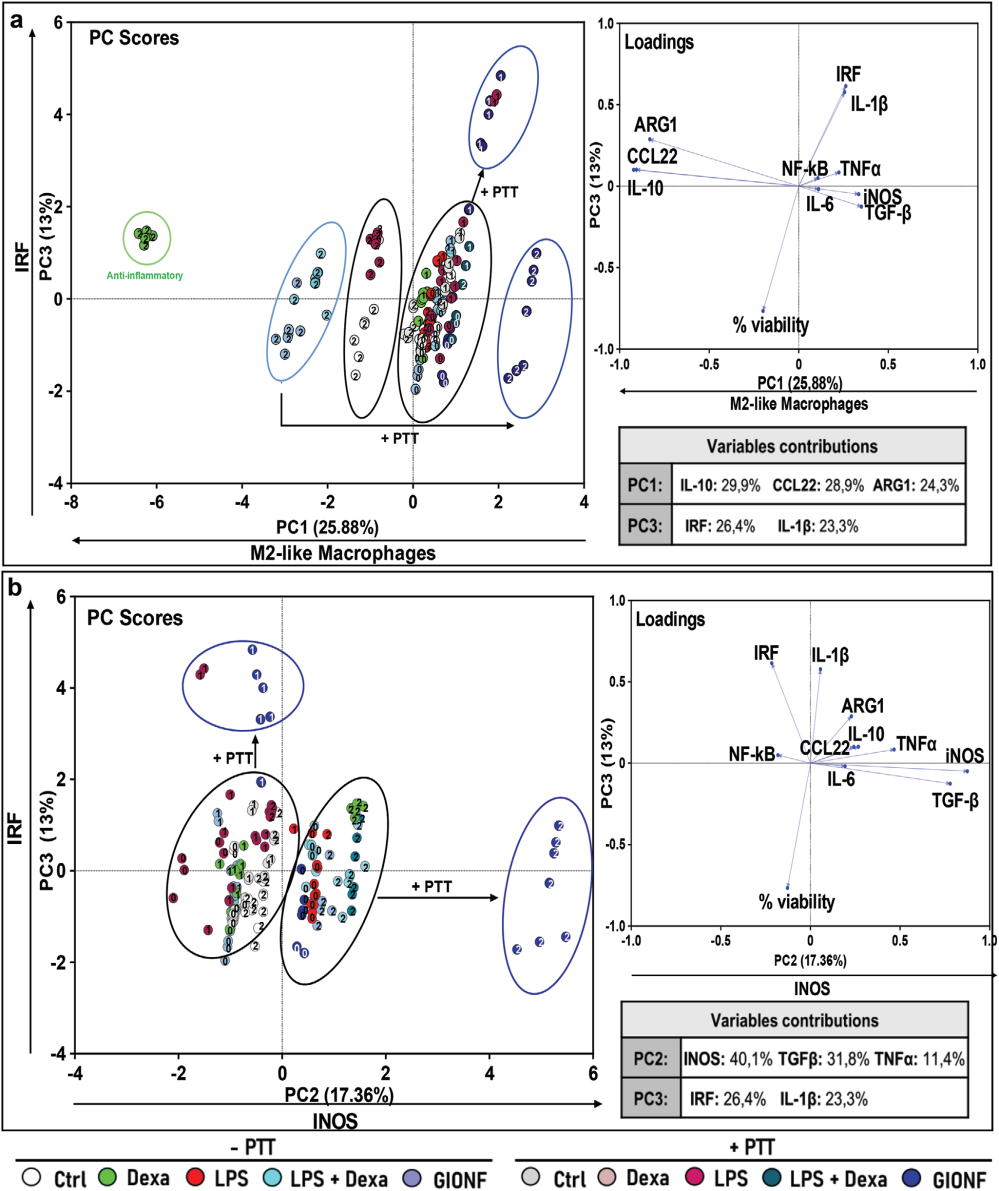
PCA of the immunomodulatory properties of GIONFs with and without PTT. a) Scatter plot of PC3 versus PC1, b) Scatter plot of PC3 versus PC2.

To better characterize the effect of GIONFs on macrophage polarization and inflammation, our screening test was used to isolated and analyze data specifically from macrophages exposed to GIONF +/− PTT and, as well as relevant controls, using PCA. This targeted approach provides a deeper understanding of the nuanced immunoreactome associated with GIONF treatments. With these restricted data, the first three principal components are PC1, a polarization gradient to M2‐like macrophages, PC2, mostly representing iNOS and TGF‐β activation and PC3, representing IRF pathway activation (**Figure** **6**).

As previously observed, M2 treated with GIONF without PTT share the same next cluster as M2 treated with dexamethasone + LPS, demonstrating the pro‐M2 effect of GIONF alone compared to the non‐treated M2. However, this M2 polarization induced by GIONF is observed without any activation of IRF pathway on PC3 (unlike treatment with dexamethasone, LPS or both that induce a basal level of IRF activation). In contrast, iNOS and TGF‐β are overexpressed during M2 polarization by GIONFs (right shift on PC2). GIONF have negligeable effect on M1 and M0 macrophages in absence of PTT.

As shown before in comparison to the other NPs, light activation on GIONFs has drastic consequences both on M1 and M2 macrophages, but in different directions. M2 treated with GIONF and PTT are at the extreme right along PC1 axis, showing M2 depolarization without activation of IRF, but with strong overexpression of iNOS and TGF‐β (right position on PC2). Besides, the PTT accentuates the M1 profile of M1 treated with GIONFs along with a strong activation of IRF pathway but without overexpression of iNOS. In the context of the tumor microenvironment, these results are particularly promising, as they suggest the potential of GIONF combined with PTT treatment to reprogram protumoral M2 macrophages into antitumoral and immunogenic M1. Simultaneously, M1 macrophages are pushed toward a more inflammatory state through the activation of the IRF pathway, which can be beneficial to promote a hot tumor microenvironment. Previous studies demonstrated that ferumoxytol, an iron oxide NP approved by the FDA for treating anaemia, inhibits tumor growth in mice, macrophage polarization toward an M1 phenotype, and eliciting a more inflammatory and anti‐tumoral response.^[^ ^19^ ^]^ Additionally, the M1 macrophage profile induced by ferumoxytol triggered a Fenton reaction, leading to the apoptotic death of adjacent tumor cells. This initiates a feedback loop resulting in the production of tumor necrosis factor‐alpha (TNF‐α) and nitric oxide (NO), as evidenced in this study.

The immunomodulatory outcomes of GIONF predicted in our in vitro screening model align with our previous in vivo observations in a preclinical model of triple negative breast cancer.^[^ ^22^ ^]^ GIONF were injected intratumorally, and the tumor was exposed to NIR light, generating mild hyperthermia at 43 °C for 15 min. Although PTT did not induce significant changes in tumor growth, it resulted in an increase of CD8+ T lymphocytes and proinflammatory cytokines such as TNFα, IL‐1β, and IL‐10 in the tumor contributing to the transition of a “cold” tumor devoid of T cells into a favorable proinflammatory T‐cell infiltrated anti‐tumoral TME. Importantly, the PTT strongly transformed the immune TME already shaped by GIONF, in line with our in vitro results. Combining a precisely timed immune response to GIONF and mild hyperthermia within an optimal window represents a potential adjunctive treatment for remotely modifying the TME. This approach could increase the effectiveness of immunotherapeutic strategies, including immune checkpoint inhibitors or CAR T cell therapy.

The most striking effect of GIONF is definitely their proficient effect to favour M2 polarization as evidenced by our screening test. This outcome could be interesting to test in contexts of inflammatory and auto‐immune diseases or tissue regeneration. Further investigations are also needed to understand the mechanisms by which M2 macrophages reverse into a M1‐like phenotype without IRF activation, but with activation of iNOS expression in response to GIONF and PTT, and the role of this novel subtype of M1‐like macrophages in the TME context and beyond. Moreover, it is interesting to note that these distinctive immunomodulatory properties of GIONF are not resumed by their individual components alone, IONF and Au NPs (Figure 5), suggesting that the association of gold and iron oxide is important in this process.

### 2.5 Magnetosomes CMD Immunomodulation on Macrophages

**Figure 7:**
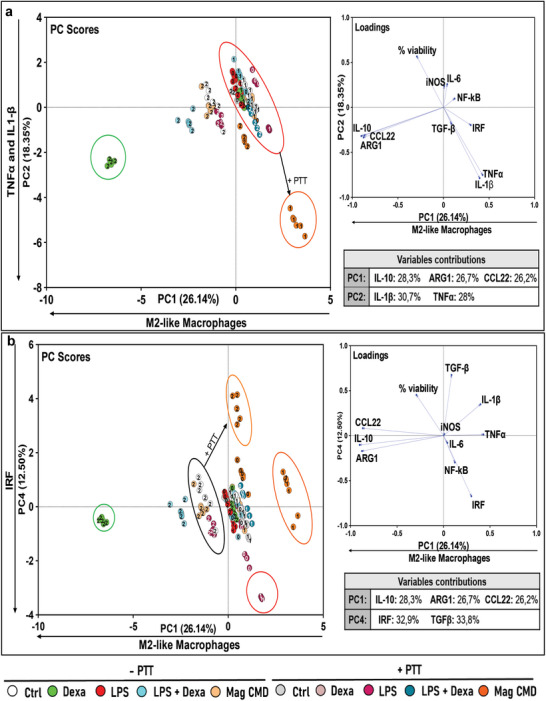
PCA of the immunomodulatory properties of magnetosomes CMD with and without PTT. a) Scatter plot of PC2 versus PC1, b) scatter plot of PC4 versus PC1.

To evaluate the immunostimulatory effects of CMD‐coated magnetosomes (CMD magnetosomes), we used our screening framework to analyze their impact with and without PTT. **Figure** **7** focuses on principal components, PC1, a polarization gradient toward M2‐like macrophages from right to left, PC2, representing the activation of TNFα and IL‐1β from top to bottom and PC4 mostly representing the activation of IRF pathway from top to bottom. In Figure 7a, without PTT, we observe that M1 treated with magnetosomes CMD belong to the cluster outlined in red close to the control M1, and to the M0, M2, and M1 treated with LPS, with or without PTT. In the absence of PTT, none of the conditions induce TNF‐α or IL‐1β activation. In this representation, the most conspicuous effect is seen on the M1 macrophages treated with CMD magnetosomes and PTT, that show a shift to the right bottom due to a strong activation of proinflammatory genes, TNFα and IL‐1β. The co‐activation of TNF‐α and IL‐1β is known to amplify the macrophage's immune response against pathogens^[^ ^41^ ^]^ and to have double‐edged sword in tumors with either pro‐oncogenic and tumor‐suppressive functions, for example by inhibiting the M2 phenotype^[^ ^42^ ^]^ or by normalizing tumor vasculature and interstitial pressure, with beneficial effects on drug delivery. When adding the PC4, we observed that this proinflammatory response to the combined treatment of magnetosomes CMD and light activation, is not dependent on IRF pathway activation. This is a significant difference with the proinflammatory response of M1 macrophages to GIONF with PTT which is activated through IRF pathway.

When looking at the M2 response, we also observed important differences between GIONFs and magnetosomes CMD. M2 cells cultured with CMD magnetosomes without PTT remain on the same cluster than the control M2, while the GIONF favor a deeper M2 polarization. Nevertheless, these M2 exposed to magnetosome CMD shift from left to right along the PC1 axis after light activation, indicating a less M2‐like profile owing to PTT and a slight activation of TNF‐α and IL‐1β. This polarization shift after PTT is also associated with TGF‐β activation and inhibition of IRF pathway as shown in Figure 7b. These results indicate that CMD magnetosomes coupled to PTT can inhibit IRF activation in M2 cell subsets but also in M1 macrophages under PTT inflammatory conditions. In contrast, GIONF strongly activate IRF pathway in M1 macrophages, but repress this pathway in M2 macrophages eliciting iNOS expression. In summary, the mechanism of action of CMD magnetosomes here passes by the initial co‐activation of TNFα and IL‐1β at the beginning of the immune response to the light exposure.

### 2.6 Gold Salt‐Induced Intracellular Aurosome Nanostructures can Modulate the Macrophages Responses and are Photoactivable by PTT

**Figure 8:**
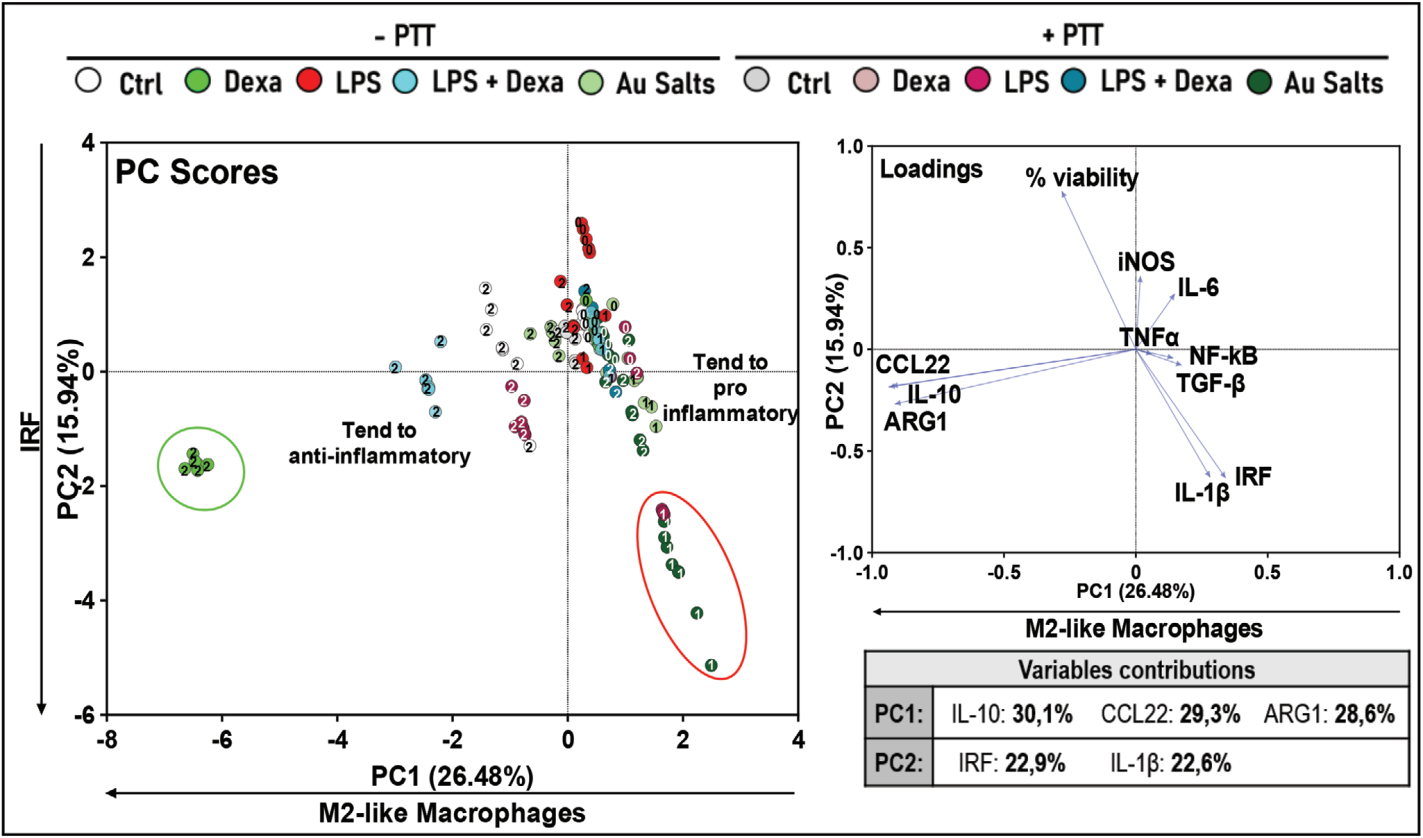
PCA of the immunomodulatory properties of gold salt treatment with and without PTT. Scatter plot of PC2 versus PC1.

Finally, we had a look on the peculiar outcome of gold salt treatment in **Figure** **8**. The two principal components were PC1, a gradient toward M2 polarization and PC2, representing the activation of IRF pathway. M0 and M1 macrophages, treated with Au salts without PTT, remain in the same cluster on the right side of the PC1 axis together with M1 controls. However, when exposed to the laser, the M1 macrophages treated with gold salt are displaced to the bottom right, demonstrating significant PTT‐inducible IL1‐β and IRF pathway activation and shift toward a more inflammatory M1 phenotype. Regarding the M2, Au salt alone displaces M2 to the right side on PC1 axis in comparison to the control. Under PTT, this right shift on PC1 is even more pronounced accompanied by an activation of IL1‐β and IRF pathway. The combined Au salt + PTT treatment thus results in a reversion of the M2 polarization and the stimulation of inflammatory M1 macrophages. Au salt forms gold aurosome nanostructures within macrophages as soon as after day 1 of treatment, which, under the impact of light exposure, induce the reprogramming of macrophages toward an inflammation state. This first confirmed the optical reactivity of aurosomes formed in macrophages, and second the potential of Au salt treatment combined with light activation to initiate a proinflammatory M1‐like response, dependent on IRF pathway. This paves the way for immunotherapeutic strategies exploiting the unique intracellular biomineralization and photosensitivity of gold salts.

### 2.7 Validation of the Multivariate Screening Model and Automatic Clustering Using Human Blood Monocyte Derived Macrophages

To enhance the robustness and broader applicability of our screening model, we extended its validation to primary macrophages derived from human blood isolated monocytes. Peripheral blood mononuclear cells (PBMCs) were isolated from blood samples of healthy donors. These monocytes were differentiated into M0 macrophages through exposure to M‐CSF or GM‐CSF and subsequently polarized into M1 and M2 phenotypes using well‐established cytokine cocktails.

Microscopic observations revealed clear morphological distinctions among the differentiated macrophages (Figure S11, Supporting Information). M0 and M2 macrophages exhibited a more elongated morphology, while M1 macrophages appeared distinctly rounder. The PCA of gene expression data provided additional validation for the differentiation process by revealing three clearly defined clusters, corresponding to M0 (blue), M1 (red), and M2 (green) macrophages, similar to the clustering observed in the THP‐1 Dual model (Figure S12, Supporting Information). We further exposed the cells to LPS and dexamethasone to evaluate the impact of these treatments on blood‐derived macrophages. PCA (Figure S13, Supporting Information) revealed that LPS drives M1 macrophages (red cluster) further to the right, intensifying their proinflammatory polarization. Moreover, M2 macrophages treated with LPS appear to shift into an M1 profile (black cluster). In contrast, dexamethasone slightly shifts the M2 macrophages (green cluster) further left along the PC1 axis. However, we found that dexamethasone‐treated M2 macrophages shared the same extended cluster as the control M2 macrophages, probably due to patient‐related variability in the immune response and suggesting that control M2 macrophages are already strongly polarized toward the M2 profile. Overall, the PCA analysis confirmed the capacity of blood derived monocyte differentiated macrophages to be polarized by classical treatments such as LPS and dexamethasone, establishing a foundation for subsequent experiments to screen the immunomodulatory effects of nanoparticles on these cells and for comparing immunoreactome responses with those observed in the THP‐1 Dual cell line.

**Figure 9:**
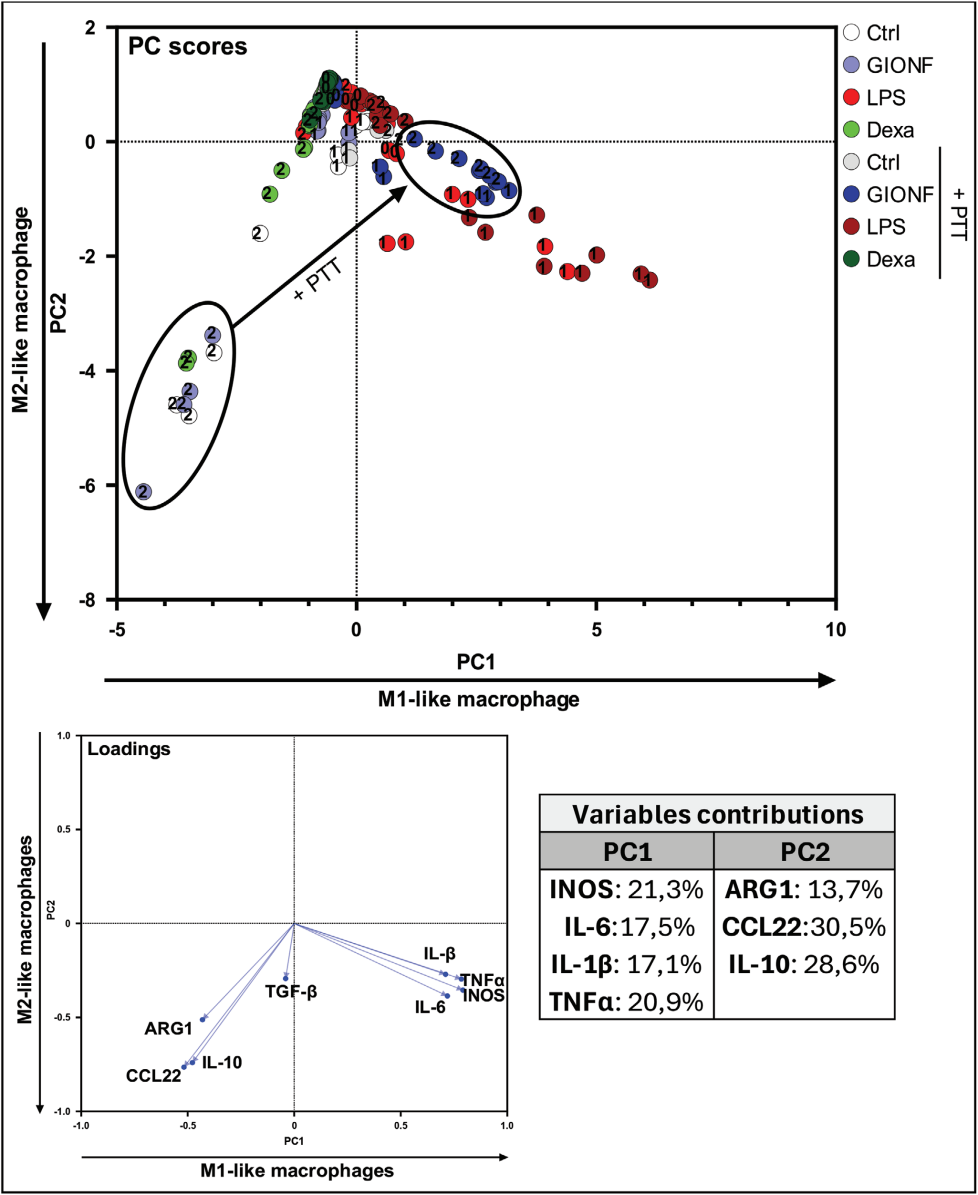
The PCA analysis illustrates the immunomodulatory properties of GIONFs, with and without laser exposure (PTT) on macrophages derived from human blood monocytes. Macrophages treated with GIONFs alone are represented in light blue, while the addition of PTT (dark blue) drives a shift in macrophage polarization from an M2‐like profile to an M1‐like profile, as indicated by a transition from the left to the right along the *x*‐axis. The experiments were performed using samples from four different donors (*N* = 4), with duplicates for each donor.

Building on this, we focused on assessing the impact of GIONFs and CMD magnetosomes, as they were identified as the most effective nanoparticles for reprogramming THP‐1 Dual macrophages. PCA on **Figure** **9** show that macrophages treated with GIONFs exhibit distinct behaviours when activated or not by the laser. M2 macrophages treated with GIONFs alone without PTT (light blue cluster) are located in the same region as the M2 control (white dot) and dexamethasone‐treated M2 macrophages (green dots), suggesting that GIONFs alone do not further enhance the M2 polarization profile. However, when M2 macrophages are treated with GIONFs and exposed to PTT (dark blue dots), a marked shift is observed from the left to the right of PC1, signifying a transition from an M2‐like profile to an M1‐like phenotype. This finding demonstrates the ability of GIONFs, combined with PTT, to successfully reprogram macrophages from an immunosuppressive M2 state toward a proinflammatory M1 state, underscoring the potential of light activated GIONF for targeted macrophage reprogramming.

**Figure 10:**
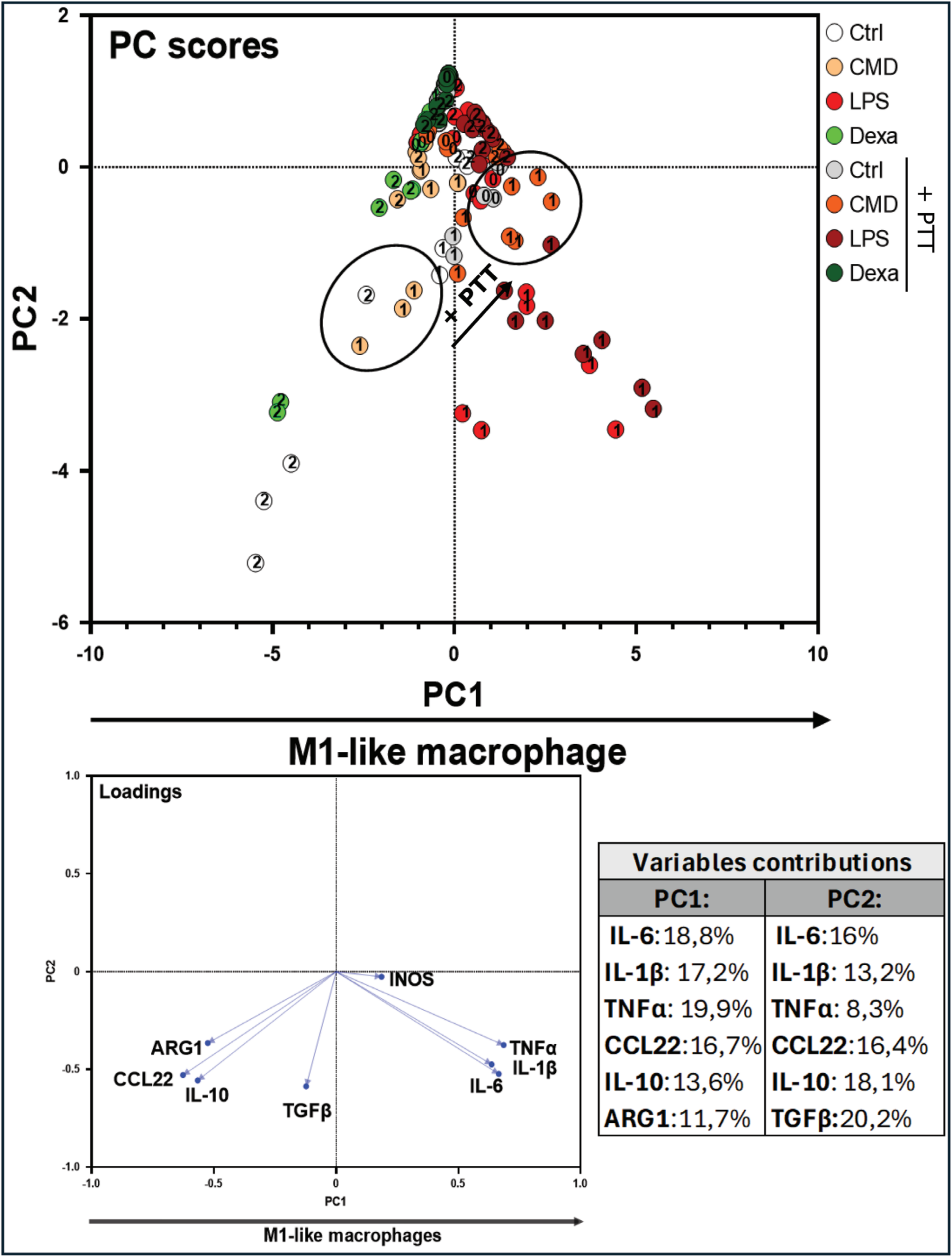
The PCA analysis highlights the immunomodulatory properties of CMD magnetosomes, with and without laser exposure (PTT), on macrophages derived from human blood monocytes. Macrophages treated with CMD magnetosomes alone (light orange) exhibit a shift from an M1‐like to an M2‐like polarization. In contrast, the addition of PTT (dark orange) shifts the macrophages to a more pronounced M1‐like profile. The experiments were performed using samples from four different donors (*N* = 4), with duplicates for each donor.

Moreover, CMD magnetosomes also exhibit significant immunomodulatory effects (**Figure** **10**). M1 macrophages treated with CMD magnetosomes per se shift leftward along PC1, suggesting a transition toward an M2‐like profile (black cluster on the left). Upon laser exposure, these macrophages return to the right, adopting a more M1‐like, proinflammatory state (black cluster to the right). This bidirectional modulation indicates that CMD magnetosomes can reprogram macrophages in response to external stimuli, such as PTT.

Interestingly, these results align with findings from the THP‐1‐Dual cell line used as a screening model, where similar macrophage reprogramming effects were observed. The validation of these effects in human blood‐derived macrophages reinforces the predictive capacity of the THP‐1‐Dual system, highlighting its utility as a robust model for assessing macrophage polarization and inflammatory responses to nanoparticle treatments. This translational relevance supports the use of THP‐1‐Dual as an effective preclinical screening tool for predicting macrophage behaviour.

## 3 Conclusion

In this study, we shed light on the complex interplay between nanoparticle exposure, light irradiation generating intracellular hyperthermia, macrophage polarization, and inflammatory state, offering insights into potential therapeutic strategies for cancer, inflammation, and autoimmune diseases. By examining with automated clustering tools, the multivariate impact of various nanoparticles on macrophage behaviour, we gained a deeper understanding of how these treatments modulate inflammation and polarization dynamics. Additionally, we established a rapid and straightforward screening test for predicting macrophage immunomodulation in vitro, which holds promise for translating the findings into in vivo applications. The first step was to validate the THP‐1‐Dual cell differentiation and polarization protocol and automated clustering in response to standard LPS and dexamethasone treatments. Importantly, the validation of this protocol was extended to macrophages derived from primary human monocytes, where the same polarization trends were observed. LPS enhanced the M1 proinflammatory profile, while dexamethasone had limited additional effects on already polarized M2 macrophages. Moreover, the effects of nanoparticles, including GIONF and magnetosomes CMD, were consistent between the primary macrophages and the THP‐1‐Dual system. GIONF, particularly when combined with photoactivation, reprogrammed M2 macrophages toward a proinflammatory M1 phenotype, while magnetosomes CMD demonstrated a similar capacity to modulate polarization dynamically and bidirectionally. These findings highlight the translational relevance of the THP‐1‐Dual model, underscoring its utility as a predictive tool for studying macrophage behaviour and immunomodulatory treatments. The concordance between the primary cell data and THP‐1‐Dual results further supports the robustness of our screening test in evaluating the immunological impact of diverse stimuli.

Moreover, this study provides a solid foundation for subsequent analyses of the complex gene expression patterns and inflammatory pathway activation in response to various nanoparticles and metallic salts with or without light exposure. Our methodology can be extended to any other stimuli, treatment, and exposure to external fields to comprehensively assess their main effects on the broad spectrum of macrophage behaviour. Moreover, any supplementary descriptors of the cellular response (cytokine release, metabolic state, morphological features…) or any other recipient cells or tissues can be added to this screening test to enrich the description of the immune response to various conditions and perform automated clustering of the immune behaviour with reduced dimensionality.

Among the demonstration of a powerful methodological approach for AI‐assisted immunological screening, our study shed light on the variety of immune responses to different nanoparticles. Furthermore, it shows that external field application, such as laser exposure, might drastically change the immunomodulatory effect of the nanoparticles and should also be evaluated in a predictive decision support test prior to in vivo experiments. By elucidating the effects of nanoparticles and metallic treatments on macrophage polarization and inflammation, we contribute to the rational design and ranking of targeted treatments aimed at modulating the tumor immune microenvironment and enhancing anti‐tumor immune responses. In a wider perspective, our study provides valuable insights into the potential of photoactivated nanoparticle as a precision timely‐ and spatially controlled strategy to shape the immune microenvironment in cancer, chronic inflammation, and auto‐immune diseases.

## 4 Experimental Section

### THP‐1‐Dual Cell Culture and Differentiation

THP‐1‐Dual cells (Sigma‐Aldrich, St. Louis, MO, USA) are a type of monocytic cell line derived from a 1‐year‐old male patient with monocytic leukaemia. These cells were seeded in 24‐well plate at 0.5 × 10^6^ cells mL^−1^ in RPMI 1640 medium (Invitrogen, 61870‐01), supplemented with 10% heat‐inactivated foetal bovine serum, 1 × 10^−3^ m sodium pyruvate (REF), 1% penicillin/streptomycin (Life technology, 15070‐063), 100 µg mL^−1^ Normocin (Invivogen, ant‐nr‐2), 10 µg mL Blasticidine (Invivogen, ant‐bl‐1), and 100 µg/mL Zeocin (Invivogen, ant‐zn‐1) at 37 °C in a 5% CO_2_ environment. To differentiate monocytes into macrophages in an M0‐like state, the cells were treated with 5 ng mL^−1^ PMA (Sigma: P1585‐5MG) for 24 h. Subsequently, the macrophages were differentiated into M1‐ or M2‐macrophages using two different cytokine cocktails for 24 h. Cocktail 1 consisted of LPS (100 ng mL^−1^) and IFN‐γ (20 ng mL^−1^) for M1 polarization, whereas cocktail 2 consisted of IL‐4 (25 ng mL^−1^) and IL‐13 (25 ng mL^−1^) for M2 polarization. After 24 h, the cells were washed 1 time with PBS1X and incubated in complete RPMI medium.

### Macrophages Derived from Human Blood Monocytes

*Isolation and Differentiation of Human Monocytes into Macrophages*: Human blood samples were obtained from healthy donors through the “Etablissement Français du Sang” (EFS) in Paris, France, under agreement #07/CABANEL/106. PBMCs were isolated by density gradient centrifugation using Lymphoprep (STEMCELL, #0 7851) from peripheral blood leukocyte separation medium. Monocytes were then enriched from the PBMC population using CD14+ magnetic beads (Miltenyi Biotec, #130‐050‐201) following the manufacturer's instructions. Isolated monocytes were cultured in RPMI‐1640 medium supplemented with 10% foetal bovine serum (FBS) at a concentration of 1 × 10⁶ cells mL^−1^.

*Differentiation into M0 Macrophages*: Monocytes were differentiated into M0 macrophages by supplementing the culture medium with either M‐CSF (50 ng mL^−1^, Miltenyi Biotec, #130‐096‐491) or GM‐CSF (50 ng mL^−1^, Miltenyi Biotec, #130‐095‐372). The cells were incubated for 7 days at 37 °C in a humidified atmosphere containing 5% CO₂. The culture medium was replaced on day 3 (D3) to maintain optimal conditions.

*Polarization into M1 Macrophages*: To induce M1 polarization, the culture medium was replaced with fresh medium containing IFN‐γ (20 ng mL^−1^) (Gibco 300–02). and LPS (100 ng mL^−1^) (Fisher Scientific:15 536 286). The cells were incubated for 24 h at 37 °C under the same culture conditions.

*Polarization into M2 Macrophages*: To induce M2 polarization, the culture medium was replaced with fresh medium containing IL‐4 (20 ng mL^−1^) (Gibco 200–04) and IL‐13 (20 ng mL^−1^) (Gibco 200–13). The cells were incubated for 24 h at 37 °C under standard culture conditions.

### Nanoparticles Synthesis

Gold Nanoparticles Au@DTDTPA: HAuCl_4_·3H_2_O (200 mg, 51 × 10^−5^ mol) was placed in a 250 mL round‐bottom flask and was dissolved with methanol (60 mL). In another flask, DTDTPA (256 mg, 50 × 10^−5^ mol), water (40 mL) and acetic acid (2 mL) were mixed. This solution containing DTDTPA was added to the gold salt solution under stirring. The mixture turned from yellow to orange. NaBH_4_ (195 mg, 515 × 10^−5^ mole) dissolved in water (13.2 mL) was added to the gold‐DTDTPA solution under stirring at room temperature. At the beginning of the NaBH_4_ addition, the solution first became dark brown then a black flocculate appeared. The vigorous stirring was maintained for 1 h before adding aqueous hydrochloric acid solution (2 mL, 1 m). After the partial removal of the solvent under reduced pressure the precipitate was retained on the polymer membrane and washed thoroughly and successively with 0.1 m hydrochloric acid, water, and acetone. The resulting black powder was dried (up to 200 mg of dry powder of Au@DTDTPA) and dispersed in aqueous solution of sodium hydroxide (NaOH 0.01 m) to have a final concentration of 50 × 10^−3^ m in gold.

Synthesis of IONF: FeCl_3_.6H_2_O (1.082 g; 4 mmol) and FeCl_2_.4H_2_O (0.398 g; 2 mmol) were completely dissolved in DEG (75 mL). The solution was stirred for 1 h. The black‐colored solution was poured with NMDEA (75 mL) and stirred again for 1 h. Separately, NaOH pellets (1.42 g; 35.6 mmol) were dissolved in a mixture of polyols (40 mL DEG and 40 mL NMDEA). This solution was added to the solution of iron chlorides and the resulting mixture was stirred for 3 h. Then, the temperature was elevated to 220 °C using a regular heating (2 °C min^−1^). Once the temperature is set to 220 °C, the solution is stirred for 4 h, and then cooled down slowly to room temperature by removing the heating plate. The black sediment was separated magnetically and washed with mixture of ethanol and ethyl acetate (1:1, v/v) for several times to eliminate organic and inorganic impurities. Possible iron hydroxides were removed by treatment with 10% nitric acid. Iron (III) nitrate (Fe(NO_3_)_3_^.^9H_2_O) (2 g, 4.9 × 10^−3^ mol) is then dissolved in water (20 mL) and added to the nanoparticles. The resulting mixture is heated to 80 °C for 45 min to achieve a complete oxidation of the nanoparticles. After another treatment with 10% nitric acid, the particles were washed twice with acetone and diethyl ether and redispersed in water. At this stage, an aqueous dispersion of maghemite nanoparticles is stable in acid or basic conditions with a point of zero charge near pH 7.3.

Synthesis of GIONF: GIONF consists of IONF decorated by Au@DTDTPA nanoparticles. The immobilization of the gold nanoparticles onto IONF requires the modification of Au@DTDTPA with dopamine.

*Modification of Au@DTDTPA with dopamine (Au@DTDTPAd)*: An aqueous solution (3 mL) containing EDC (0.207 g; 1.08 × 10^−3^ mol) and NHS (0.247 g; 2150 × 10^−3^ mol) was added to a suspension of Au@DTDTPA gold nanoparticles (6 mL, 10 g Au L^−1^). The suspension was stirred at pH 6 for 90 min. Afterwards, an aqueous solution (4 mL) containing dopamine (9.45 × 10^−3^ g; 2.25 × 10^−5^ mol) was added to the suspension under stirring at pH 7.5. The mixture is stirred overnight. The purification of the suspension of gold nanoparticles was performed by dialysis against water (MWCO: 6–8 kDa) for 12 h. Water bath was changed three times every 3 h.

*Immobilization of Au@DTDTPAd onto IONF*: Under stirring, the suspension of Au@DTDTPAd was mixed with the suspension of IONF (6 mL; 35 g Fe L^−1^). The mixture with a pH of 5.5 was heated at 50 °C for 24 h. Successive washings were performed with ultrapure water, acetone, and diethyl ether until a clear supernatant was obtained. After purification the iron oxide nanoflowers decorated with gold nanoparticles (GIONF) were introduced in ultrapure water in order to provide an aqueous suspension of GIONF.

Synthesis of Magnetosomes: MSR‐1 bacteria were cultured in fermenters with precise control over environmental parameters, including maintaining the temperature at 29.5 °C and pH at 6.9 under microaerobic conditions. Following cultivation, the cells were washed, concentrated by tangential flow filtration, and frozen at −80 °C. Magnetosomes were extracted through alkaline lysis followed by several washes. After 48 h of freezing, the bacteria were thawed and heated to 80 °C in a water bath and then lysed at an optical density of 20 (565 nm) with a final concentration of 2 m potassium hydroxide. Magnetosomes were isolated via magnetic selection and washed three times with 10× PBS buffer to adjust the pH to 7, and three times with deionized water to remove excess salts. The magnetosome samples were centrifuged at 4000 rpm at 10 °C for 15 min, the supernatant was discarded, and the nanoparticles were frozen at −80 °C for a minimum of 48 h before lyophilization. Frozen magnetosomes were lyophilized for 24 h at −50 °C and a pressure of 0.015 mBar using a Labconco freeze‐dryer (Labconco, Kansas City, USA). The lyophilized samples were then ground into a fine powder using an agate mortar to reduce aggregates and achieve a homogeneous powder. To purify magnetosomes, 1 gm of magnetosomes was placed in a crucible and heated in a muffle furnace with a temperature increase of 6 °C min^−1^ up to 420 °C, with stages at 50 °C for 1 h, 270 °C for 2 h, 310 °C for 2 h, and finally 420 °C for 2 h. The purified magnetosomes were then analyzed by CHNS spectroscopy to determine the residual organic material content. Following this analysis, the magnetosomes were weighed and suspended in a sterile apyrogenic CMD (carboxymethyl dextran) or CA (Citric acid) solution at a concentration of 200 mg mL^−1^. This suspension was sonicated for 15 h in an ultrasonic bath at a frequency of 25 kHz and a pH of 4.5 to promote the formation of the CMD or CA‐magnetosome complex. Then, the magnetosomes were centrifuged at 4000 rpm for 1 h at 8 °C and washed with filtered apyrogenic water to remove any remaining impurities. Finally, magnetosoms were formulated before lyophilisation. For CMD coated magnetosomes D‐Sorbitol (1 g) was dissolved in ultrapure water to a final volume of 10 mL then vortexed thoroughly. The solution was filtered using a 0.22 µm syringe filter into a clean conical tube and stored at 4 °C. CMD coated magnetosomes were centrifuged at 14 500 rpm for 20 min at room temperature. The supernatant was discarded, and the pellet was resuspended in 100 µL of 10% sorbitol solution for each Eppendorf tube, ensuring that 10 mg of iron was present per tube. For CA coated 50 µL of 15% sucrose solution was added to each tube ensuring the drop reached the magnetosomes at the bottom. Similarly, 50 µL of 5% PEG 4000 solution was added. The tubes were then vortexed for 10 s and samples were sonicated for 10 min at 25 kHz and 100% power. Samples were frozen in liquid nitrogen for 10 min and lyophilized at −50 °C and 0.024 mBar for 18 h. Post‐lyophilization and before using magnetosomes for experiments, 1 mL of ultrapure water was added in the Eppendorf tube, and the samples were stored at 4 °C.

Synthesis of RCL: The RCL nanoparticles were provided by Resonant Circuits Limited as a dispersion of multicore magnetic NPs in an isotonic solution of water for injection and saline. The constituent dextran‐coated iron oxide nanoparticles were formed as a result of a reaction, at ≈65 °C, of pharmaceutical grade iron salts and low‐molecular weight (40 kDa) dextran, mediated by the addition of a reducing agent.

Au Salts: Sodium aurothiomalate powder (Product No. 157201‐1G, Sigma‐Aldrich) was used to prepare the solution. The powder was diluted in ultrapure water to achieve a concentration of 10 mg mL^−1^. To ensure complete dissolution, the mixture was gently stirred until the gold powder was fully dissolved, ensuring a homogeneous solution. The solution was then filtered at 0.22 µm and the prepared solution was stored at 4 °C and protected from light to maintain stability until further use.

Treatments on Cells. After differentiation, cells were incubated for 24 h with different NPs or gold salt suspension at a concentration 5 µg mL^−1^ of iron or gold in RPMI complete medium. LPS (100 ng mL^−1^) and dexamethasone (Dexa, 10 µg mL^−1^) were used as positive and negative controls for inflammation, respectively. These two controls were added at the same time as the NPs treatment. After treatment, the cells were washed 1 time with PBS 1× and incubate in complete RMPI medium.

Photothermal Therapy (PTT). The cell monolayer were exposed to near infrared irradiation (NIR) 808 nm optical fiber laser (laser model F‐808‐3 W; Optical fiber: 400 µm/0.22NA, PhotonTec Berlin GmbH) at 1 W cm^−2^ for 10 min. Photothermal heating profiles of nanoparticles in RPMI culture medium under NIR irradiation was acquired using a thermal camera Flir T430sc (see Supporting Information S10) as described.^[^ ^43^ ^]^

NF‐kB and IRF Monitoring with THP‐1‐Dual Macrophages. For NF‐κB pathway detection, SEAP secreted in the medium was measured using 180 µL of QUANTI‐Blue solution (rep‐qbs1) detection reagents (preparation followed by the manufacturer's instructions) mixed with 20 µL of cell supernatant for each well. SEAP secretion in the supernatant induces a turn of the purple/pink QUANTI‐Blue solution to blue and allows indirect monitoring of NF‐κB pathway activation by measuring absorbance at 620–655 nm on a spectrometer. For the IRF pathway detection, luciferase production was measured using luciferin. Luminescence detection was performed using 50 µL of QUANTI‑Luc (rep‐qlc1) solution detection reagent mixed with 10 µL of cells supernatant for each well. LPS (100 ng mL^−1^) was used as a positive control to activate the IRF and NF‐κB pathways, and dexamethasone (10 µg mL^−1^) was used as a negative control to reduce the activation of both pathways. luminescence was then read using a spectrophotometer.

Macrophage Polarization Confirmation by RT‐qPCR. Total RNA was extracted from lysed cells using a RNeasy Mini Kit (Qiagen, #74 136). The quantity and quality of RNA were measured using a NanoDrop spectrometer. Reverse transcription was performed with 500 ng of each RNA sample using SuperScript II Reverse Transcriptase (Invitrogen, 18064‐014) and RNAase (Promega, N251B). The qPCR reaction was performed with PowerUp SYBR Green Master Mix (Applied Biosystems, #A25742) and primers from Eurogentec (**Table** **1**) using the following thermal cycles: one step of 2 min at 95 °C and 40 cycles of 15 s at 95 °C, followed by 1 min at 60 °C. RPLP0 was used as a reference gene for normalization. mRNA levels were quantified using the threshold cycle method.

**Table 1:** qPCR oligonucleotides used for macrophages polarization from Eurogentec.

qPCR primers		
Genes	Fwd	Rev
**INOS**	CCCCTTCAATGGCTGGTACA	GCGCTGGACGTCACAGAA
**IL‐6**	CGGG AACGAAAGAGAA GCTCTA	GGCGCT TGTGGA GAAGGA
**IL‐1β**	TCAGCCAATCTTCATTGCTCAA	TGGCGAGCTCAGGTACTTCTG
**CCL22**	CGGCGCCAACATGGAA	CAGACGGTAACGGACGTAATCA
**TGFβ**	CGCGCATCCTAGACCCTTT	CTGTGGCAGGTCGGAGAGA
**RPLP0 **	TGCATCAGTACCCCATTCTATCAT	AAGGTGTAATCCGTCTCCACAGA
**TNFα**	TCAATCGGCCCGACTATCTC	CAGGGCAATGATCCCAAAGT
**ARG1**	GCGCCAAGTCCAGAACCA	CGTGGCTGTCCCTTTGAGAA
**IL‐10**	TGAGAACAGCTGCACCCACTT	TCGGAGATCTCGAAGCATGTTA

Alamar Blue. Cellular metabolic activity was quantified using the redox indicator resazurin in the form of the Alamar Blue assay. THP‐1‐Dual‐derived macrophages (M0, M1, and M2 phenotypes) were plated at a density of 5 × 10^5^ cells per well in an opaque 24‐well plate. Triton‐treated cells (0.3%) served as a negative control, while untreated cells acted as a positive control. Nanoparticles were incubated for 24 h in complete RPMI medium, and a PTT session was applied if necessary. Twenty hours later the Alamar Blue reagent (Invitrogen, Carlsbad, CA, USA) was prepared in a 1:10 dilution with RPMI medium devoid of phenol red and supplemented with 1% penicillin/streptomycin. Subsequently, 200 µL of this diluted Alamar Blue solution was added to each well. The incubation period lasted for 3–4 h at 37 °C, ceasing once the solution in the positive control wells turned pink, indicating metabolic activity. Fluorescence quantification was performed using a microplate reader with an excitation of 560 nm and emission of 590 nm. To calculate the cell viability, blank readings from wells with only medium and Alamar Blue were deducted from those of the test wells, and the resultant percentage reduction was computed using the provided formula from the manufacturer, reflecting a direct relationship between fluorescence intensity and cell viability.

Human Cytokine Quantification: The MSD multiplex cytokine assay (reference K15049D) served as a rapid sandwich immunoassay method for quantifying cytokines levels in the supernatants. Supernatant samples were securely stored at −80 °C until required for cytokine measurements. Leveraging the principles of sandwich enzyme‐linked immunosorbent assay (ELISA), the mesoscale multiplexing immunoassay employs commercially available capture. Antibodies were precoated on the conductive plates onto which the samples were applied. The concentrations of interferon gamma (IFN‐γ), interleukin IL‐1β, IL‐2, IL‐4, IL‐10, IL‐12p70, and IL‐13, were quantified using MESO QuickPlex SQ 120 mm system and data were analyzed using MSD DISCOVERY WORKBENCH analysis software, providing a comprehensive assessment of the cytokine profile.

Transmission Electron Microscopy: Cells were fixed with 2% glutaraldehyde (Sigma‐Aldrich) in a 0.1 mol L^−1^ sodium cacodylate buffer (Sigma Aldrich) for 1 h at ambient temperature, washed with cacodylate buffer (0.2 M), and kept in this buffer at 4 °C until inclusion. Cell was then contrasted with Oolong Tea Extract (0.5% in cacodylate buffer), osmium tetroxyde (1% in cacodylate buffer), and potassium cyanoferrate (1.5% in cacodylate buffer). They were then gradually dehydrated in ethanol (25 to 100%). The samples were then substituted gradually in propylene oxide, a mix of propylene oxide and Epon, and finally, embedded in pure Epon (Delta microscopie, Labège, France). Thin sections (70 nm) of samples were collected on 200‐mesh grids. Observations were performed on a Hitachi HT7700 microscope (Platform Microscopie et Imagerie des micro‐organismes, animaux et aliments 2, UMR1313, INRAE /AgroParis Tech, Jouy‐en‐Josas, France).

Nanoparticles TEM images were obtained with a JEOL 2100+ microscope (Laboratoire Materiaux et Phenomenes Quantiques, UMR7162, CNRS Université Paris Cité, Paris, France) operating at 200 kV after deposition of a droplet of NPs solution on a hydrophilized 300‐mesh grid.

### Statistical Analysis

All results are expressed and plot as mean ± standard error of the mean SEM. Univariate statistical analyses were performed using an unpaired *t*‐test followed by Kolmogorov–Smirnov test in nonparametric conditions. Differences among multiple groups were tested using one‐way ANOVA followed by Holm‐Sidak post‐test or Kruskall–Wallis in nonparametric conditions. Significant differences between groups are expressed as follows: ^*^ *p* < 0.05, ^**^ *p* < 0.01, or ^***^ *p* < 0.001. Multivariate statistical analysis was performed using PCA with GraphPad Prism 10.1.2 software (San Diego, CA, USA). PCA allowed to display automatically unsupervised data clustering.

## Conflict of Interest

The remaining authors declare that the research was conducted in the absence of any commercial or financial relationships that could be construed as a potential conflict of interest.

## Supporting information



Supporting Information

## Data Availability

The data that support the findings of this study are available in the supplementary material of this article.
