# Supplementary material for: Multivariate Screening and Automated Clustering of Macrophage Immunoreactome to Nanoparticles and Photothermal Therapy
Source: Adv Sci (Weinh). 2025 Jun 26;12(31):2405860. doi: 10.1002/advs.202405860 (PMC12376647; doi:10.1002/advs.202405860)
Supplement: Supplementary file 1 — Supporting Information [file ADVS-12-2405860-s001.pdf]

ADVANCED SCIENCE
Open Access

# Supporting Information

for *Adv. Sci.*, DOI 10.1002/advs.202405860

Multivariate Screening and Automated Clustering of Macrophage Immunoreactome to Nanoparticles and Photothermal Therapy

*Sonia Becharef, Léa Jabbour, Nassima Bekaddour, Giulio Avveduto, Nathalie Luciani, Gautier Laurent, Rana Bazzi, Edouard Alphandery, Stéphane Roux, Amanda K. A. Silva, Kelly Aubertin, Jean-Philippe Herbeuval* and Florence Gazeau**

# Supplementary materials

## Multivariate Screening and Automated Clustering of Macrophage Immunoreactome to Nanoparticles and Photothermal Therapy

*Sonia Becharef*[1], *Léa Jabbour*[1], *Nassima Bekaddour*[4], *Giulio Avvedutto*[1], *Nathalie Luciani*[1], *Gautier Laurent*[3], *Rana Bazzi*[3] *Edouard Alphandery*[2], *Stéphane Roux*[3], *Amanda K. A. Silva*[1], *Kelly Aubertin*[1], *Jean-Philippe Herbeuval*[4]*, *Florence Gazeau*[1]*

[1]Université Paris Cité, NABI, CNRS UMR8175, INSERM U1334 Paris, France

[2]Nanobacterie, Paris, France

[3]Université de Franche-Comté, CNRS, Chrono-environnement, F-25000, Besançon, France

[4] Université Paris Cité, LCBPT CNRS, UMR8601, Team Chemistry & Biology, Modeling & Immunology for Therapy, Paris, France.

Corresponding authors: florence.gazeau@u-paris.fr, jean-philippe.herbeuval@parisdescartes.fr

Keywords: macrophages, nanoparticles, photothermal therapy, immunotherapy

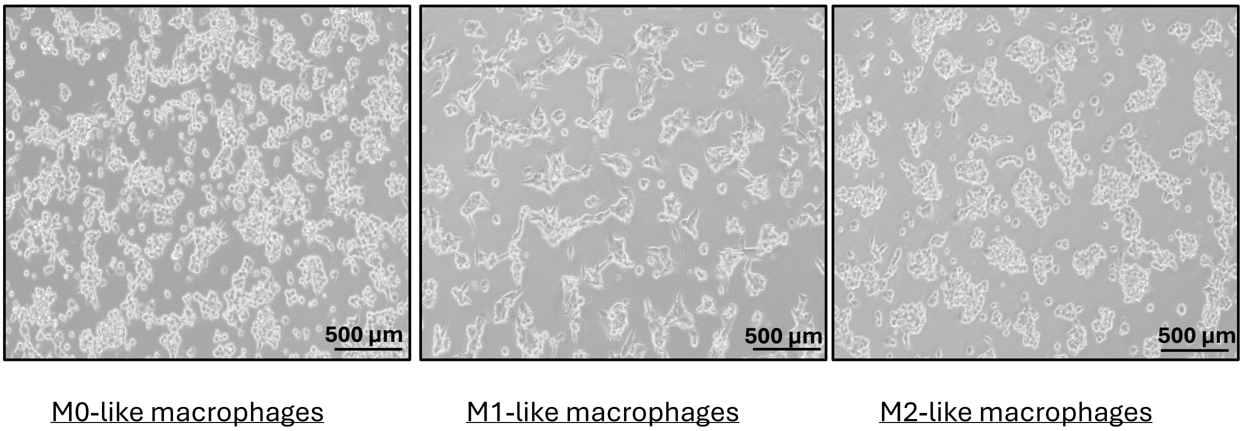


**Figure S1:** Representative microscopy pictures of THP-1 Dual® macrophages differentiated and polarized using the protocol established in this study. The images capture distinct morphologies of macrophages corresponding to the different M0, M1 and M2 polarization states. These images were acquired using the EVOS microscope (Thermofisher) under Brightfield channel illumination.

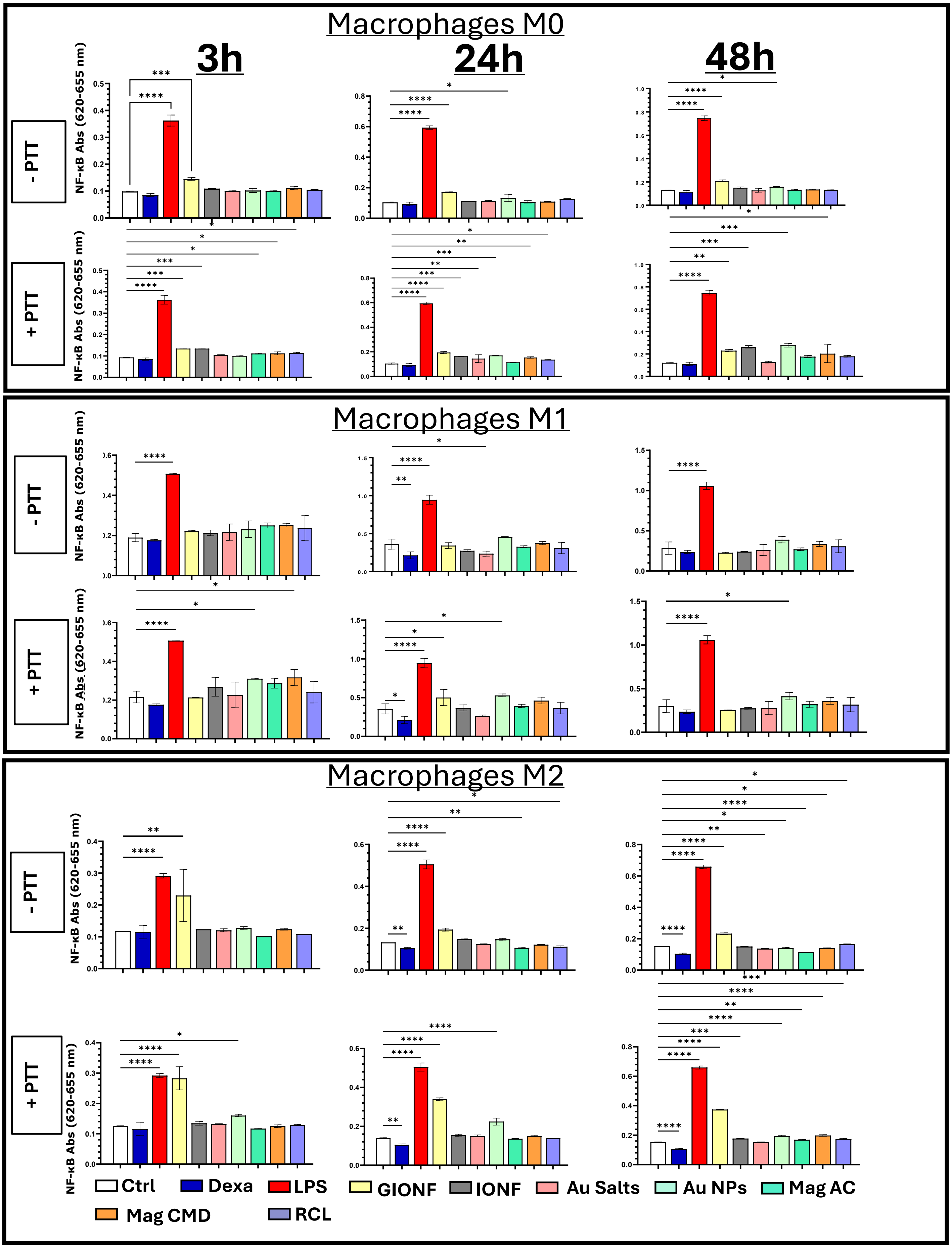
Macrophages M0
3h
24h
48h
- PTT
+ PTT
Macrophages M1
- PTT
+ PTT
Macrophages M2
- PTT
+ PTT
NF-κB Abs (620-655 nm)
Ctrl
Dexa
LPS
GIONF
IONF
Au Salts
Au NPs
Mag AC
Mag CMD
RCL

**Figure S2:** Monitoring the activation of NF-κB pathways activation in macrophage M0, M1, and M2 by measuring absorbance (Abs) at 620-655 nm during 3 time points (3h, 24h and 48h) upon exposure to nanoparticles (NPs) and photothermal therapy (PTT), to track the dynamic change during treatment. A pharmacological anti-inflammatory agent, dexamethasone, and a pro-inflammatory stimulus, lipopolysaccharide (LPS), serve as controls. Additionally, a combination control of dexamethasone and LPS is included to assess the protective impact of dexamethasone under inflammatory conditions, particularly its role in modulating the NF-κB pathway activation. Results are shown as the mean ± S.D, N=3 and results of univariate statistical analysis are illustrated by *, ** or ***, respectively indicating $p < 0.05$, 0.01 or 0.001.

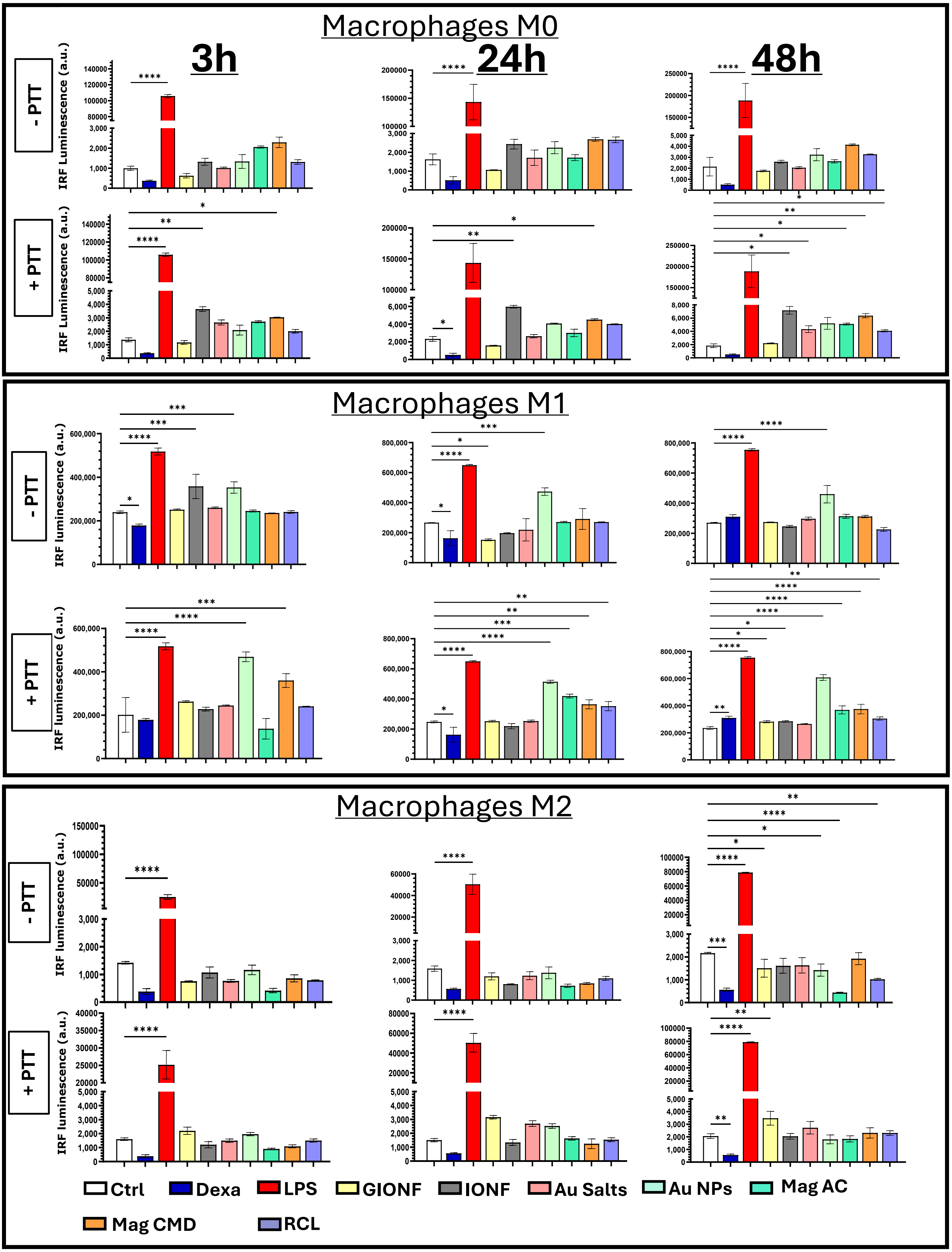
Macrophages M0
3h
24h
48h
- PTT
+ PTT
IRF Luminescence (a.u.)
Macrophages M1
IRF luminescence (a.u.)
Macrophages M2
Ctrl
Dexa
LPS
GIONF
IONF
Au Salts
Au NPs
Mag AC
Mag CMD
RCL

**Figure S3:** Monitoring the activation of IRF pathways in macrophage M0, M1, and M2 by measuring luminescence (a.u= arbitrary unit) during 3 times point (3h, 24h and 48h) upon exposure to nanoparticles (NPs) and photothermal therapy (PTT), to track the dynamic change during treatment. A pharmacological anti-inflammatory agent, dexamethasone, and a pro-inflammatory stimulus, lipopolysaccharide (LPS), serve as controls. Additionally, a combination control of dexamethasone and LPS is included to assess the protective impact of dexamethasone under inflammatory conditions, particularly its role in modulating the activation of the IRF pathways. Results are shown as the mean ± S.D. N=3 and results of univariate statistical analysis are illustrated by *, ** or ***, respectively indicating p < 0.05, 0.01 or 0.001.

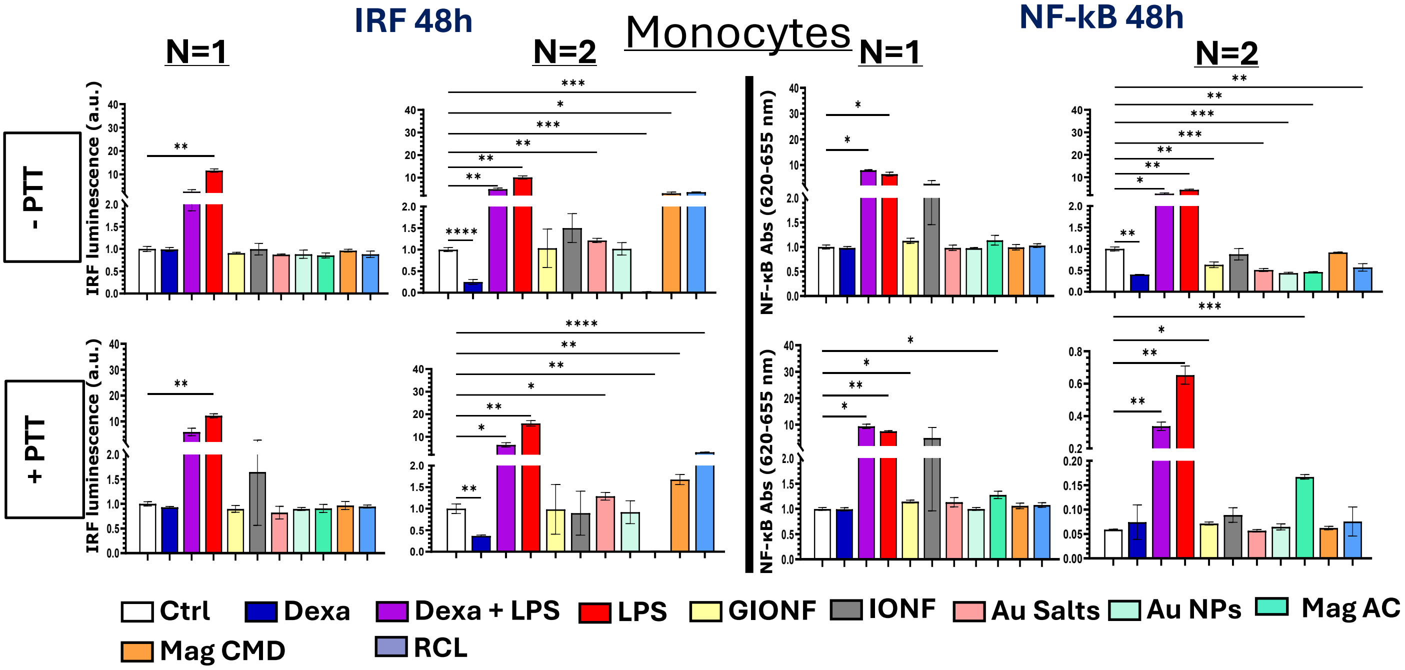


**Figure S4**: Monitoring the activation of IRF and NF-κB pathways in monocytes by measuring luminescence (a.u.= arbitrary unit) and absorbance (Abs) at 620-655 nm respectively during 3 times point (3h, 24h and 48h) upon exposure to nanoparticles (NPs) and photothermal therapy (PTT), to track the dynamic change during treatment. A pharmacological anti-inflammatory agent, dexamethasone, and a pro-inflammatory stimulus, lipopolysaccharide (LPS), serve as controls. Additionally, a combination control of dexamethasone and LPS is included to assess the protective impact of dexamethasone under inflammatory conditions, particularly its role in modulating the activation of the aforementioned pathways. Results are shown as the mean ± 1 S.D. N=3 and results of univariate statistical analysis are illustrated by *, ** or ***, respectively indicating p < 0.05, 0.01 or 0.001.

# Aurosomes day 7

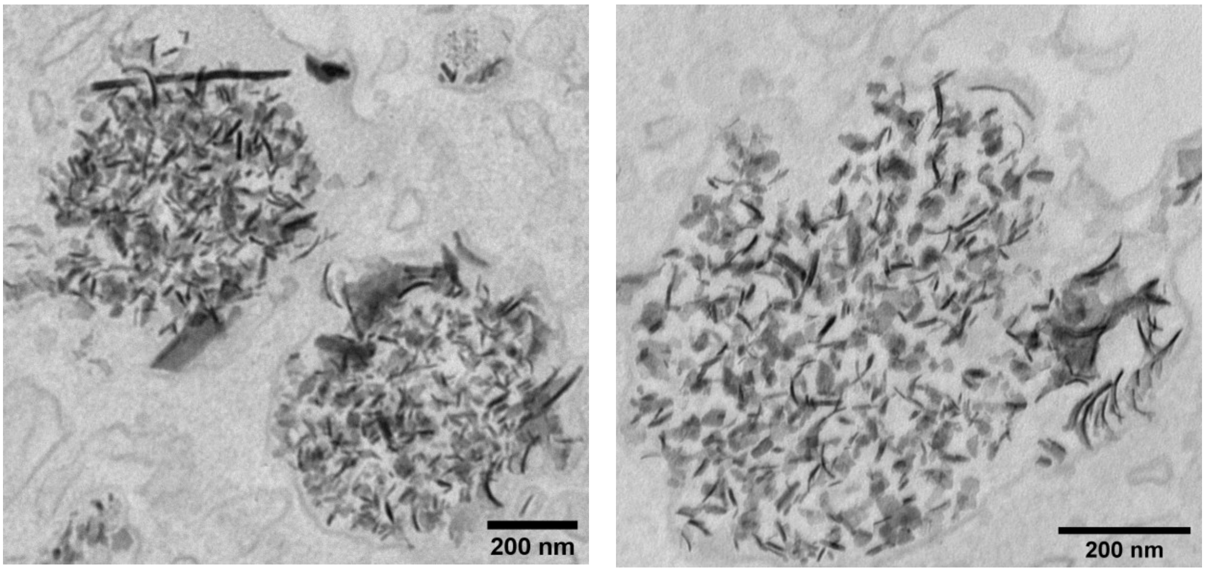


**Figure S5**: TEM images of intracellular gold nanostructures (aurosomes) in M0 macrophages at day 7 after treatment with Au salt. M0 macrophages were treated with Au Salts (39 µg/mL) for 24h and rinsed, cultured for 6 further days before glutaraldehyde fixation, resin inclusion, slicing and TEM observation.

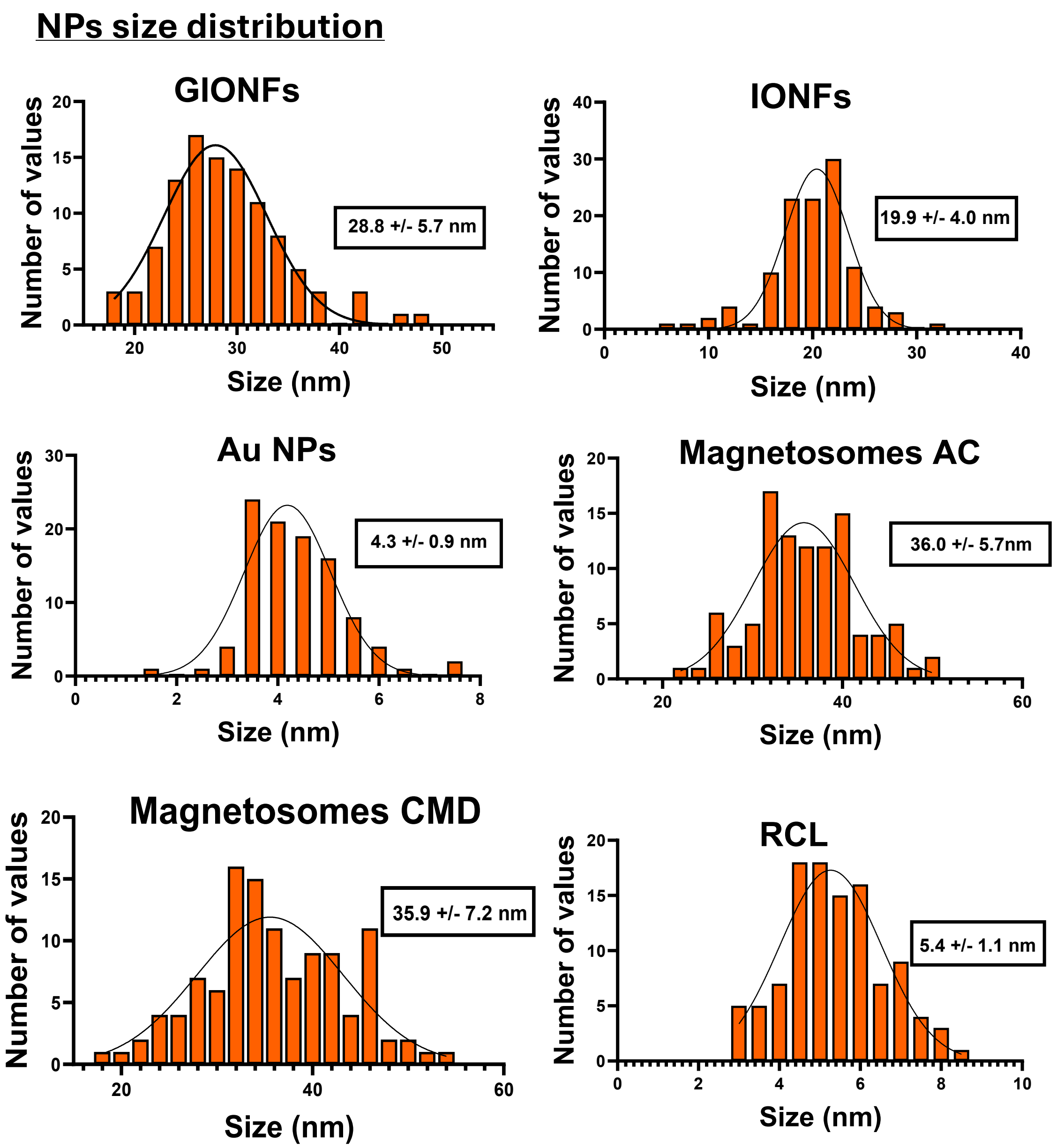


**Figure S6**: Size distribution of the different nanoparticles used in this study. The distributions were quantified through the measurement of nanoparticle size from transmission electron microscopy (TEM) images. A minimum of 100 nanoparticles were analyzed to ascertain this distribution. The mean size ± Standard Deviation is indicated in insert.

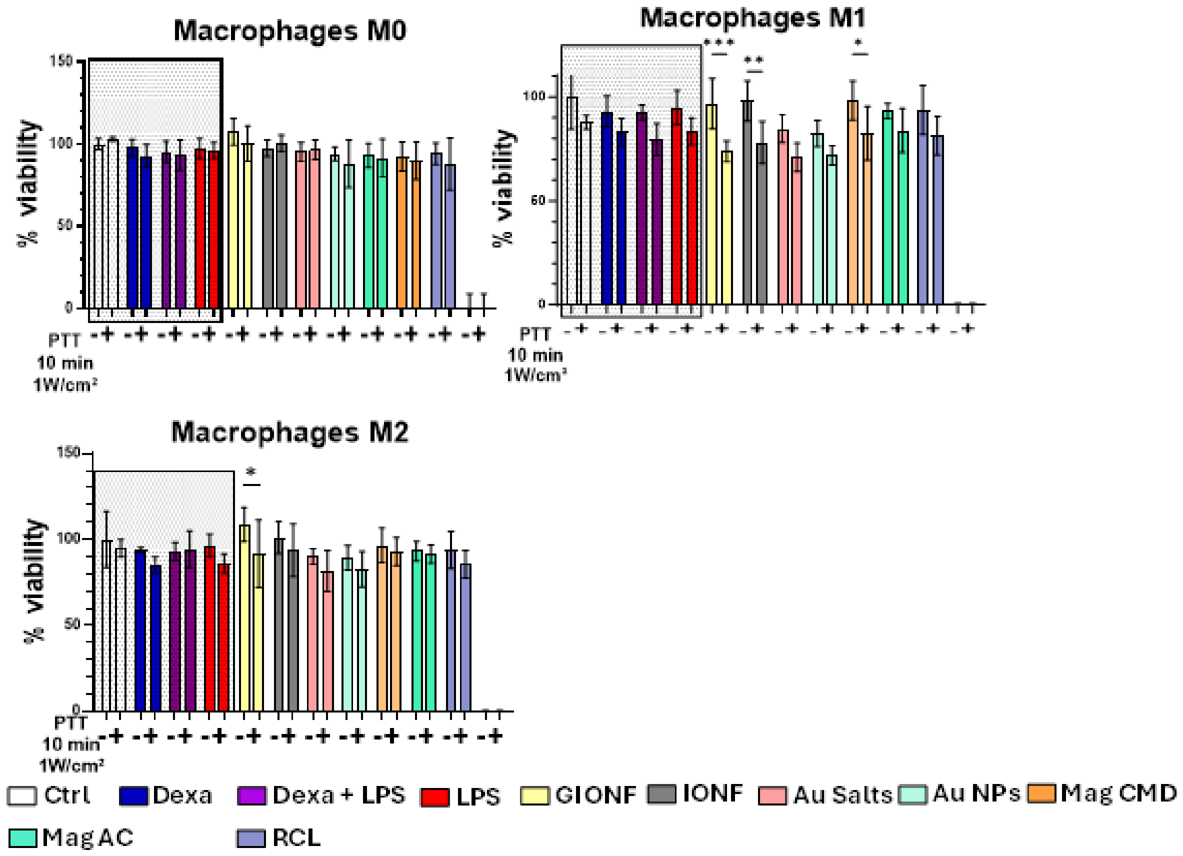


**Figure S7: Mitochondrial metabolic activity (Alamar Blue Assay) of THP-1 Dual® cells differentiated and polarized into M0, M1, and M2 Macrophages exposed to various treatments with and without light exposure (PTT).** 24 hours after differentiation/polarization protocol, cells were treated with NPs or Au salts for 24 hours. Subsequently, conditions marked '+ PTT' were exposed to photothermal therapy (PTT) for 10 minutes using 808 nm light at an intensity of 1W/cm². Cells were then carefully washed before performing Alamar Blue assay to assess the mitochondrial activity of the cells. Percentage of viability was calculated as the mitochondrial metabolic activity of the treated cells normalized by the metabolic activity of the non-treated cells (Control PBS without PTT). It was observed that NPs at a concentration of 5µg/ml did not induce cellular toxicity, maintaining cell viability above 80% which is acceptable based on ISO 10993-5 standard. The addition of PTT at this subtoxic concentration resulted in a slight reduction in cell viability, which did not fall below 70%, indicating that the NPs were effectively activated by PTT causing a cellular death at the edge of acceptance for ISO standard. This allows for the assessment of immunomodulatory effects potentially due to the combined treatment rather than to cytotoxicity. Results are shown as the mean ± S.D. and performed on three independent experiments (N=3). Statistical analysis was carried out with a Two-way ANOVA test followed by a Sidak post-test. *, ** or ***: Indicates that the result is statistically different from the control respectively with $p < 0.05$, 0.01 or 0.001. GraphPad Prism 10.1.2 software (San Diego, CA, USA) was used for statistical analysis and PCA.

# M1 gene expression marker in macrophages

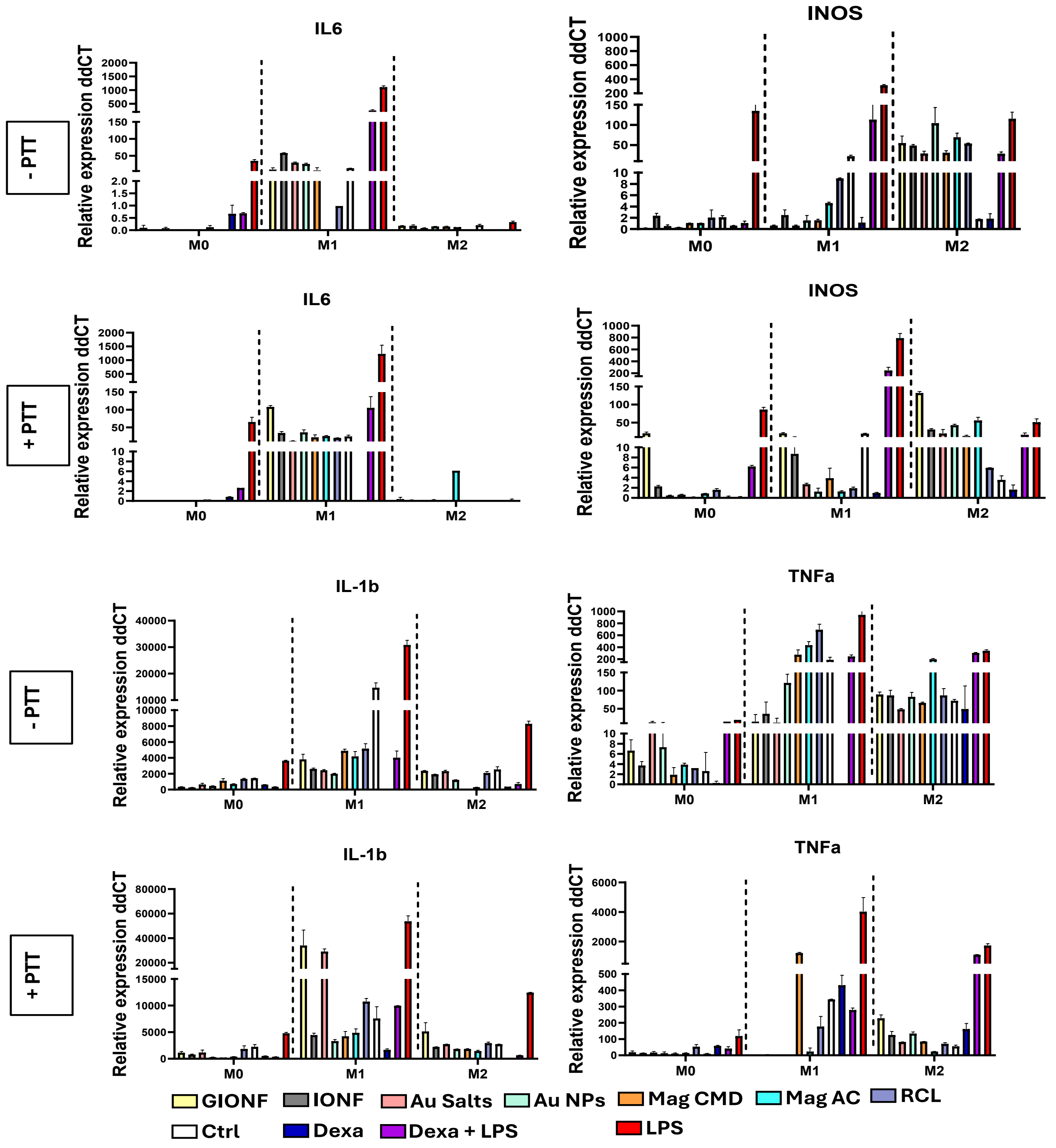


**Figure S8: Nanoparticle and Photothermal Therapy Effects on M1 Marker Gene Expression in THP-1 Dual® Derived M0, M1 and M2 Macrophages.** This figure quantifies the impact of NPs and PTT on the transcriptional landscape of genes associated with the M1 macrophage phenotypes. Results confirm that M1 markers are more expressed in M1 macrophages and suggest that nanoparticle exposure, coupled with photothermal therapy, selectively modulates gene expression, predominantly enhancing M1 marker genes while the effects on M2 markers are less pronounced, implying a shift towards a pro-inflammatory macrophage profile. Gene expression levels were normalized to the housekeeping gene RPLP0. Results are shown as the mean ± S.D. based on an experimental N=2. Significance levels from univariate statistical analyses are denoted as follows: * for $p < 0.05$, ** for $p < 0.01$, and *** for $p < 0.001$, respectively.

# M2 gene expression marker in macrophages

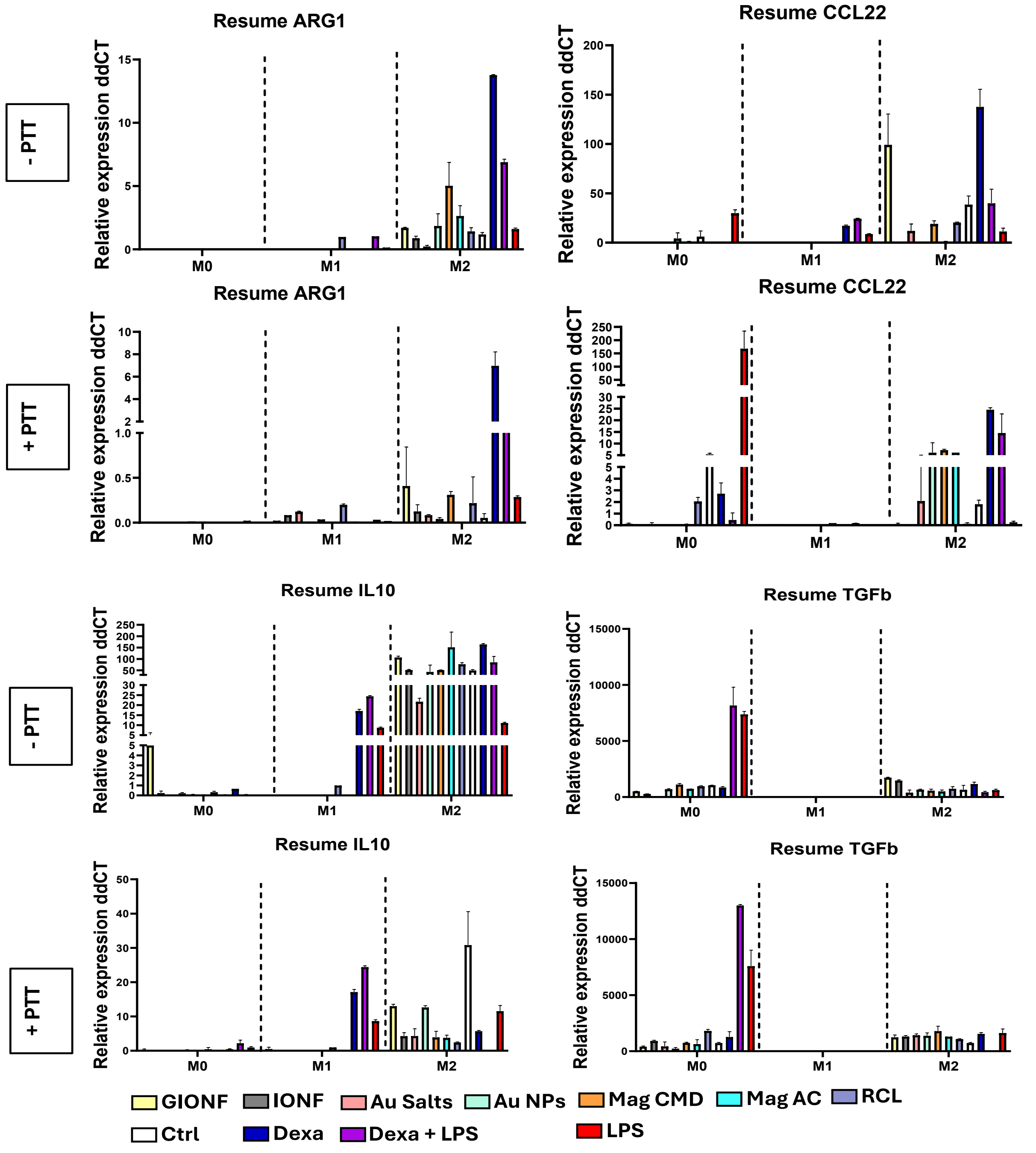


**Figure S9: Nanoparticle and Photothermal Therapy Effects on M2 Marker Gene Expression in THP-1 Dual® Derived M0, M1 and M2 Macrophages**. This figure quantifies the impact of NPs and PTT on the transcriptional landscape of genes associated with the M2 macrophage phenotypes. Results confirm that M2 markers are more expressed in M2 macrophages and suggest that nanoparticle exposure, coupled with photothermal therapy, selectively down modulates M2 gene expression due to a more inflammatory condition. Gene expression levels were normalized to the housekeeping gene RPLP0. Results are shown as the mean ± S.D. based on an experimental N=2. Significance levels from univariate statistical analyses are denoted as follows: * for $p < 0.05$, ** for $p < 0.01$, and *** for $p < 0.001$, respectively.

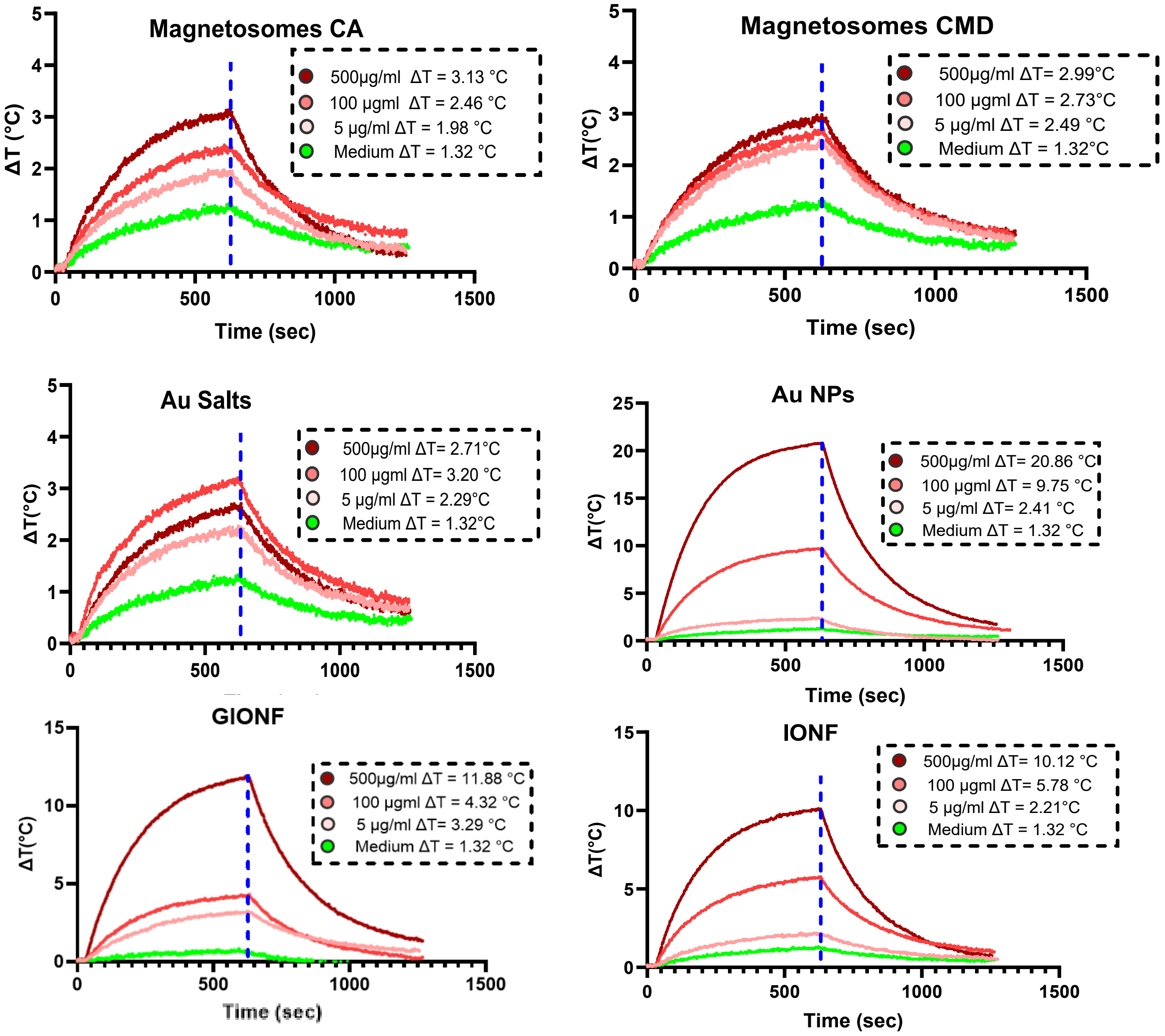


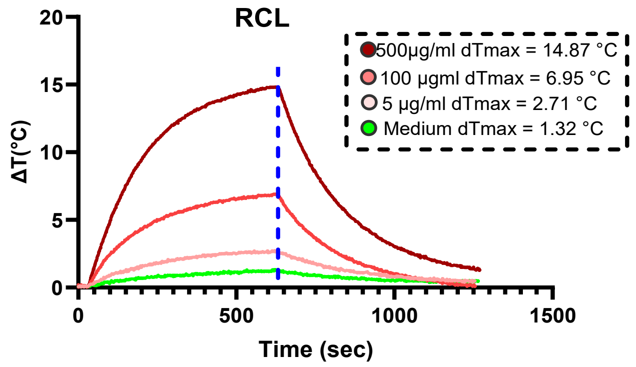


1. $\Delta T = \Delta T_m \cdot k$

2. $k = (\Delta T^n_{d1}) / (\Delta T^n_{dx})$

**Figure S10: Photothermal heating profiles of nanoparticles in RPMI Culture Medium under NIR irradiation**. This figure presents the photothermal response of three different concentrations of nanoparticles (500, 100, and 5 µg/ml) dispersed in RPMI culture medium and of the RPMI medium only in a final volume of 200 µl. Samples were subjected to 808 nm laser exposure (1 W/cm2, PTT) for 10 minutes as described in [1] and the resulting delta temperature ($\Delta T_m$) were recorded using a thermal camera (Flir T430sc, FLIR Systems) at the bottom of Eppendorf tubes. After the laser was turned off, temperature changes were monitored for an additional 10 minutes to observe the cooling phase without PTT. $\Delta T_m$ curves were measured at different times in a controlled room. To avoid errors due to temperature fluctuations at different days all data have been multiplied by a correction coefficient "k" which is needed to compare and standardize the temperature measurement performed at different conditions. This coefficient consists in the ratio between the reference $\Delta T^n_{d1}$ of nigrosine (Nigrosine, 198285, Sigma-Aldrich, St. Louis, MO, USA) taken at the first laser exposure and the $\Delta T^n_{dx}$ of the same solution taken the day of each laser exposures. Hence showed in the figure are the final $\Delta T = \Delta T_m \cdot k$. [1]. As a matter of fact, nigrosin uniquely converts all absorbed energy into heat with a thermal conversion efficiency of 100%, making it an ideal standard for comparing the heating effects observed with NPs samples at different conditions. This way is possible to minimize possible effects due to the environmental differences.

The thermal camera's output reveals minimal culture medium heating at the 5 µg/ml nanoparticles concentration compared to the medium control. This indicates that nanoparticle-mediated immunomodulatory and macrophage polarization effects are likely due to localized cellular heating due to intracellular NPs rather than any thermal alteration of the surrounding culture medium due to NPs that could be still present in the culture medium after carefully rinsing of the cell monolayers.

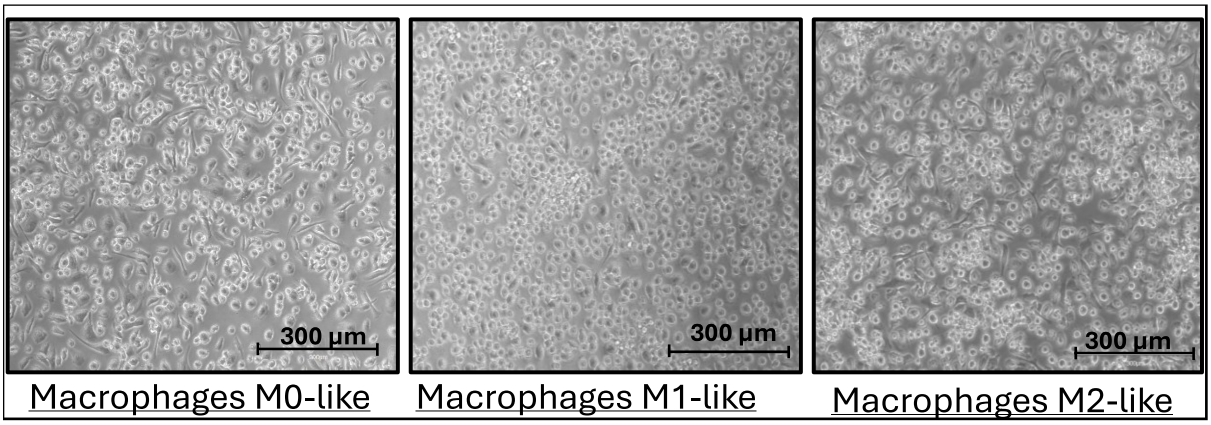


**Figure S11:** Representative microscopy pictures of macrophages derived from human blood monocyte differentiated and polarized in M0, M1 and M2 profile. The images capture distinct morphologies of macrophages corresponding to the different M0, M1 and M2 polarization states. These images were acquired using the EVOS microscope (Thermofisher) under Brightfield channel illumination.

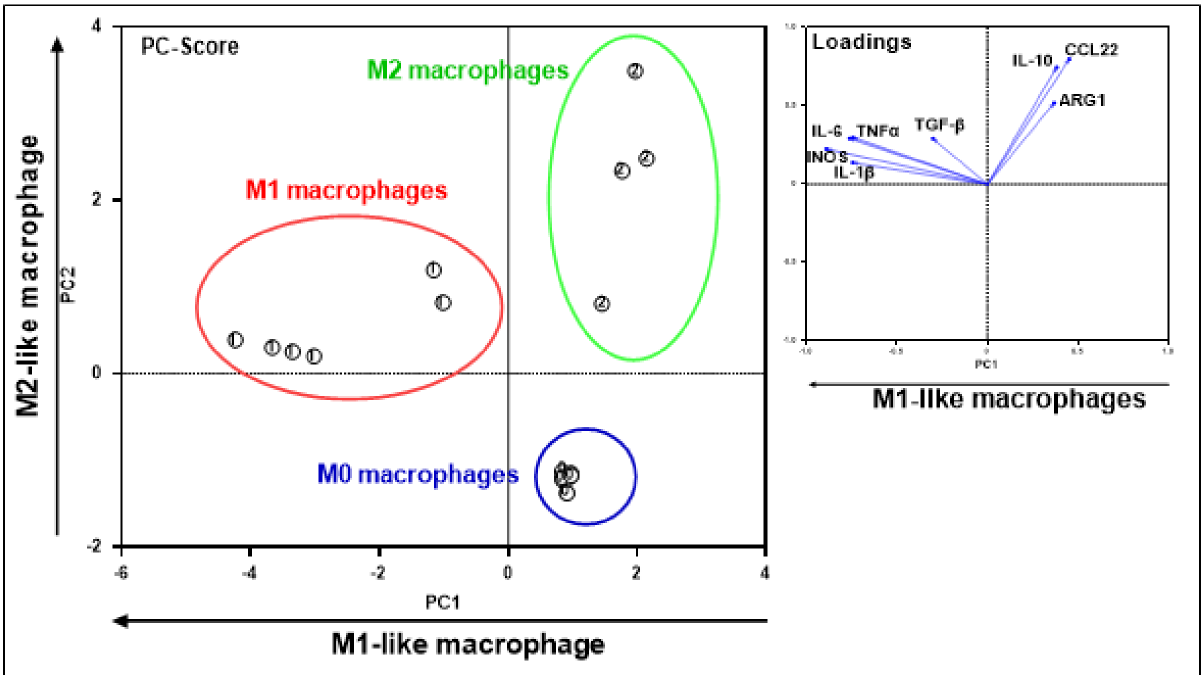


| Variables contributions | | | | |
|---|---|---|---|---|
| **PC1:** | **INOS**: 25,9% | **IL-6**: 19% | **IL-1β**: 18,2% | **TNFα**: 18,1% |
| **PC2:** | **ARG1**: 15% | **CCL22**: 35,7% | **IL-10**: 31% | |

**Figure S12:** Validation of the differentiation and polarization process of macrophages derived from primary human blood monocytes into M0, M1, and M2 phenotypes. PCA was conducted on these differentiated macrophages, revealing three distinct clusters corresponding to M0 (blue), M1 (red), and M2 (green) macrophages on the PCA scatter plot (PC scores, left). This clustering confirms the robustness and reliability of our differentiation protocol. The experiments were performed using samples from four different donors (N=4), with duplicates conducted for each donor.

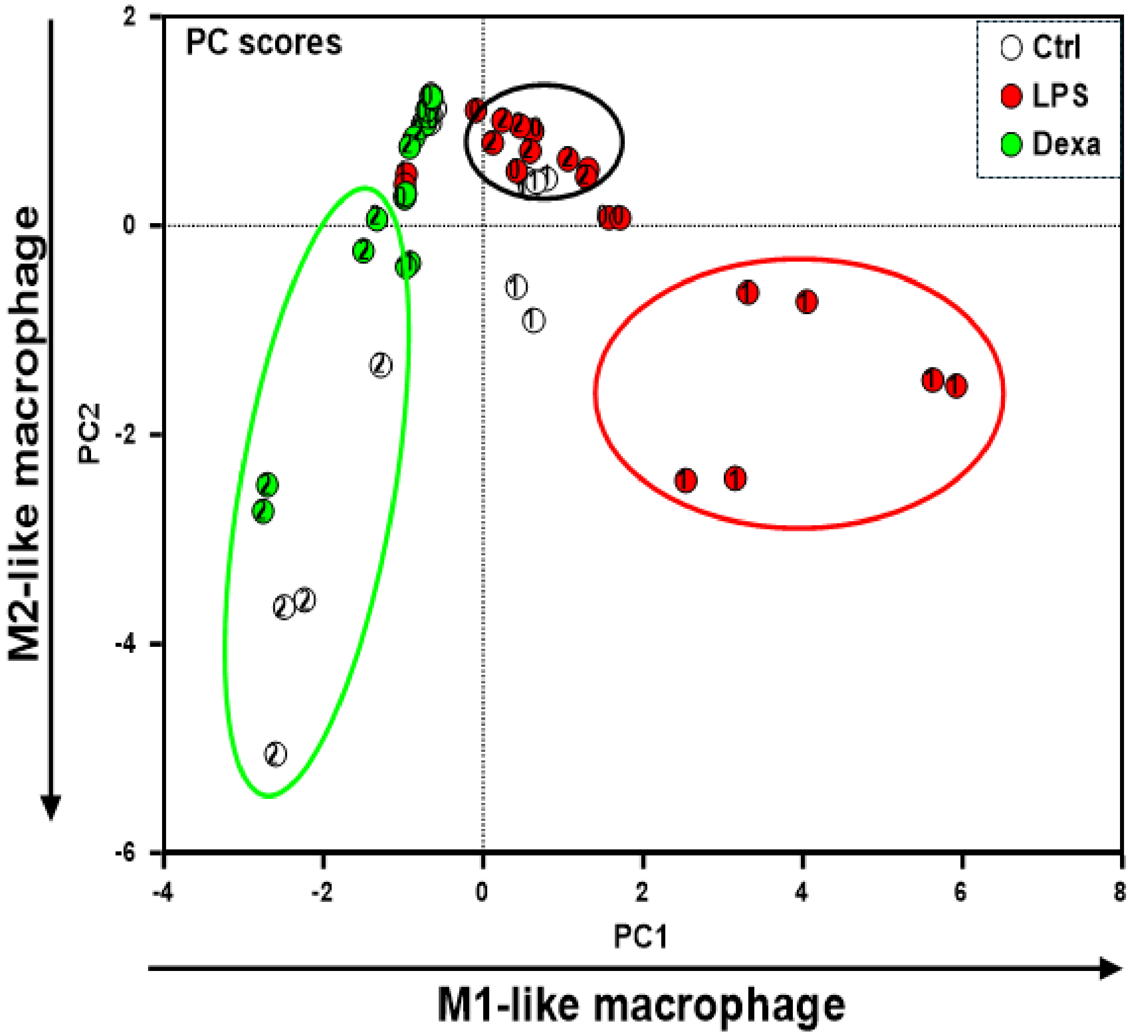


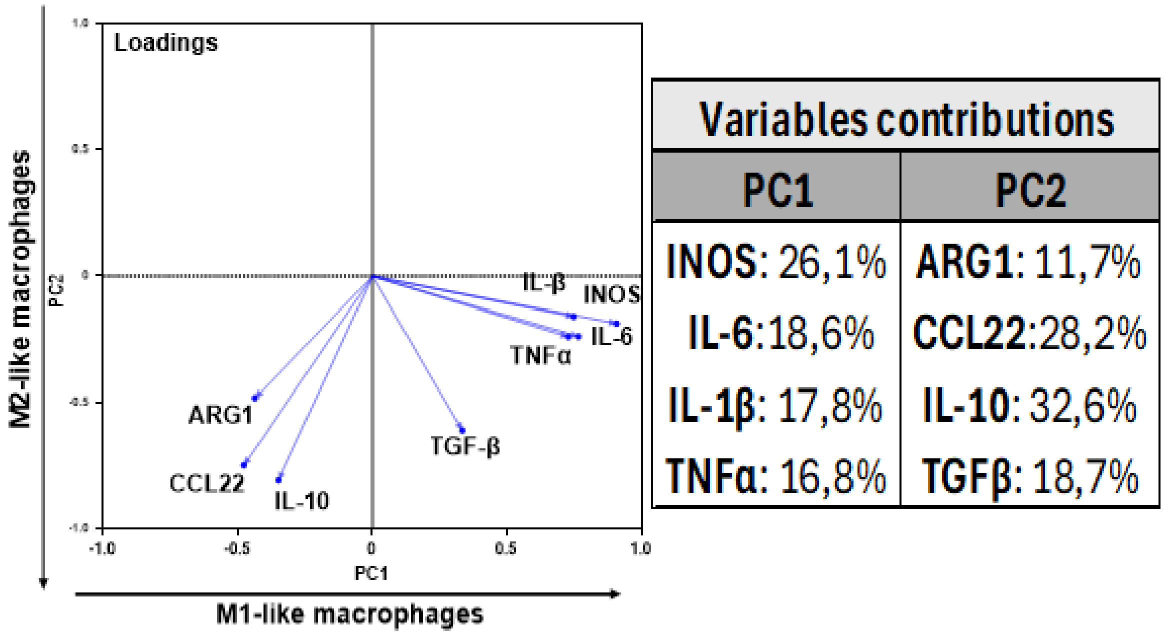


| Variables contributions | |
|---|---|
| **PC1** | **PC2** |
| **INOS**: 26,1% | **ARG1**: 11,7% |
| **IL-6**:18,6% | **CCL22**:28,2% |
| **IL-1β**: 17,8% | **IL-10**: 32,6% |
| **TNFα**: 16,8% | **TGFβ**: 18,7% |

**Figure S13*:*** Principal Component Analysis (PCA) of macrophage derived from human blood monocytes polarization following treatments. PC1 (horizontal axis) represents the gradient of polarisation to M1-like macrophages profile, while PC2 (vertical axis) captures the degree of M2-like macrophages polarization. Control macrophages (white dot) show clear separation, with M1 macrophages clustering on the right and M2 macrophages on the left. LPS treatment (red) intensifies the M1 pro-inflammatory polarization and shifts M2 macrophages towards an M1-like profile. In contrast, dexamethasone treatment (green) minimally affects M2 macrophages, which remain strongly polarized towards the M2 profile. These results demonstrate the potential for immunomodulation of macrophages through external treatments. The experiments were performed using samples from four different donors (N=4), with duplicates for each donor.

**Bibliography**:

1. Lucas, T. *et al.* Quantitative, precise and multi-wavelength evaluation of the light-to-heat conversion efficiency for nanoparticular photothermal agents with calibrated photoacoustic spectroscopy. *Nanoscale* **15**, 17085–17096 (2023).
